# Land sparing or sharing: A paired stream study to evaluate the relationship between land use configuration and macroinvertebrate diversity in streams

**DOI:** 10.1371/journal.pone.0354515

**Published:** 2026-08-12

**Authors:** Sugjit Singh Padda, Bradley J. Cardinale

**Affiliations:** Department of Ecosystem Science and Management, The Pennsylvania State University, University Park, Pennsylvania, United States of America; University of Zagreb Faculty of Teacher Education, CROATIA

## Abstract

Increasing food production often comes at the cost of habitat loss, which contributes to declining biodiversity. Consequently, balancing global food production with biodiversity conservation is a growing challenge, particularly as agricultural production expands and intensifies to meet the needs of a growing human population. The land sharing–land sparing (LSLS) framework presents two contrasting strategies for managing the trade-off between food production and biodiversity conservation. Land sharing involves low-intensity agriculture being interspersed within heterogeneous landscapes that retain natural habitats, whereas land sparing concentrates high-yield agriculture in some areas to allow separate areas to remain undisturbed as natural habitat designated for conservation. While LSLS has been studied extensively in terrestrial ecosystems, its relevance for freshwater ecosystems, despite their exceptional biodiversity and vulnerability, remains largely unexplored. Here, we evaluate how aquatic macroinvertebrate diversity is influenced by LSLS land use configurations in agricultural landscapes of Pennsylvania, USA. We compared macroinvertebrate community metrics (taxa richness, evenness, total abundance), and community composition between paired stream reaches—one flowing through a land sharing landscape configuration and the other through a land sparing configuration. The paired study design ensured streams were matched for physical habitat characteristics and most water quality variables known to influence macroinvertebrates, thus allowing us to isolate any potential impacts of land configuration *per se*. We found no significant differences in analyzed macroinvertebrate community metrics or composition between paired streams flowing through land sharing versus land sparing configurations. The only differences identified were small changes in the abundance of collector-filterers that are likely to be biologically insignificant. As one of the first empirical tests of the LSLS framework in aquatic ecosystems, these findings suggest that the spatial configuration of agricultural land use, whether integrated with or spatially separated from natural habitat, may have limited influence on macroinvertebrate diversity in streams, cautioning against overgeneralizing terrestrial conservation strategies for use in freshwater ecosystems.

## Introduction

To sustain a rapidly growing global population, the Food and Agriculture Organization (FAO) estimates that agricultural systems will need to increase their output by nearly 50% compared to 2012 levels—encompassing not only food, but also fiber and biofuel production [1–2]. Conversion of land to agricultural habitat is the primary driver of global biodiversity loss. Indeed, the Intergovernmental Science-Policy Platform on Biodiversity and Ecosystem Services IPBES (2019) identified land-use change, alongside direct exploitation of nature (e.g., logging, hunting, and harvesting), as responsible for over half of all human impacts on terrestrial and freshwater biodiversity [3–4]. Land-use change alone is projected to imperil more than 1,700 species by 2070 [5], and the potential biodiversity loss is greatest in freshwater ecosystems, which are losing species at rates that are two to three times higher than terrestrial ecosystems [6–7]. The need for agricultural expansion to support a growing human population directly challenges the goals of international conservation efforts, such as the Kunming-Montreal Global Biodiversity Framework that seeks to protect 30% of land and water by 2030 [8].

Two contrasting land-use strategies have been proposed to better balance the dual objectives of food production and biodiversity conservation: land sharing and land sparing. Land sharing, originally termed ‘wildlife-friendly farming’, refers to low-yield or low-intensity agricultural practices interspersed within heterogeneous landscapes that include more natural or semi-natural habitats that serve as reservoirs for biodiversity [9–12]. The land sharing approach encompasses systems such as silvopastoral and integrated farming [13–14]. Conversely, land sparing involves the spatial separation of high-intensity agricultural habitat from contiguous natural areas that are of higher value for conservation [12,14,15]. The two land management strategies differ in two regards: (1) landscape configuration—land sharing systems intersperse agricultural with natural habitats, while land sparing systems separate the two habitat types spatially;—and (2) agricultural intensity — land sparing is typically associated with higher-intensity management aimed at maximizing yields on reduced land area whereas land sharing is often claimed to have less intense farming practices.

To date, studies looking at how land sharing–land sparing (LSLS) configurations influence biodiversity have mostly been performed in terrestrial ecosystems, including forests, grasslands, and riparian habitats, and across a range of agricultural land uses such as crop cultivation, grazing, and timber extraction [12,16–19]. As studies on land sharing and land sparing have proliferated, empirical studies have provided mixed support for both strategies in promoting biodiversity conservation, with some even advocating for hybrid approaches that integrate elements of both within a given landscape [20–22]. In contrast to empirical work, modeling studies have more consistently favored land sparing [20–22]. Even so, the debate over which strategy is superior remains unresolved [23].

While terrestrial studies of the LSLS framework have addressed the role of land use configuration, their implications for aquatic ecosystems, such as streams and rivers that flow through different landscape configurations, remain largely unexplored [23]. The lack of work on freshwater systems is a key research gap given that freshwater ecosystems support approximately half of all fish species and over 100,000 macroinvertebrate taxa despite covering less than 1% of the Earth’s surface [24–27]. Previous studies have made clear that aquatic species diversity is impacted by agricultural land use at both local (e.g., stream reach) and regional (e.g., watershed) scales and is often negatively associated with agricultural intensity [28–29]. Even so, few (if any) studies have assessed how the spatial configuration of agricultural and more natural habitats in the surrounding watershed might influence biodiversity in streams [29]. The spatial configuration of agricultural and natural habitats has potential to influence biodiversity by affecting the dispersal and reproductive success of the aerial adult stage of stream macroinvertebrates [30–31]. Variation in terrestrial land-use configuration has been linked to differences in macroinvertebrate species composition, abundance, and richness [30,32,33], as well as increased community segregation associated with landscapes that have greater habitat heterogeneity [34]. As such, there is good reason to hypothesize that macroinvertebrate species diversity and/or community composition might differ among land sparing vs. sharing landscapes irrespective of the intensity of agriculture in those landscapes.

Here we report results from a paired stream study that investigated how land sharing and land sparing spatial configurations influence stream macroinvertebrate biodiversity in the state of Pennsylvania, USA. We compared macroinvertebrate community metrics (taxa richness, evenness, abundance), community composition, and the abundance of functional feeding groups (FFGs) between paired stream reaches, one of which ran through a watershed that was characterized by land sharing, and a second comparable partner stream that ran through a land sparing configuration. As we will show, the paired design allowed us to hold many physical and chemical variables that are known to influence freshwater macroinvertebrates constant, while isolating any potential effects of spatial land use configuration *per se*.

## Methods

### Site selection

We selected 36 stream pairs (72 streams total), where each pair was located within the same ecoregion and watershed. All study sites were in central and western Pennsylvania, USA, within the Appalachian region. Site pairs were distributed across three U.S. EPA Level III ecoregions (67, 69, and 70), which span the Ridge and Valley, Western Allegheny Plateau, and Central Appalachians [35–36]. In each pair, one stream flowed through a landscape where agricultural land was interspersed with forest (i.e., land sharing), while the other flowed through a landscape where agriculture and forest were more spatially separated (i.e., land sparing). To ensure comparability, we selected streams that were of similar size (i.e., stream order, width, and depth) within the same watershed and ecoregion, as described below:

To identify suitable sites, we used GIS data layers including: U.S. EPA Level III ecoregions (defined by geology, climate, soils, and vegetation), HUC-8 subbasins (medium-sized hydrologic units, the HUC is a hydrologic unit code system used by USGS to identify hydrologic areas), stream networks in Pennsylvania (from the National Hydrography Plus High Resolution Dataset), and stream access points (e.g., bridges) from the Pennsylvania Spatial Data Access portal. We selected Level III ecoregions for their ecological specificity relative to Levels I and II, while still being broad enough to encompass multiple HUC-8 subbasins. HUC-8 subbasins were chosen for their manageable size, which supports replication across ecoregions while capturing ecological and biodiversity variability. Our site selection criteria follow previous studies that have used HUC-8 watersheds to explore ecological patterns across geographic regions [36–37]. Subbasins were included if at least 50% of their area overlapped a target ecoregion, contained both agricultural and forested land, and had at least 100 stream access points.

**Fig 1:**
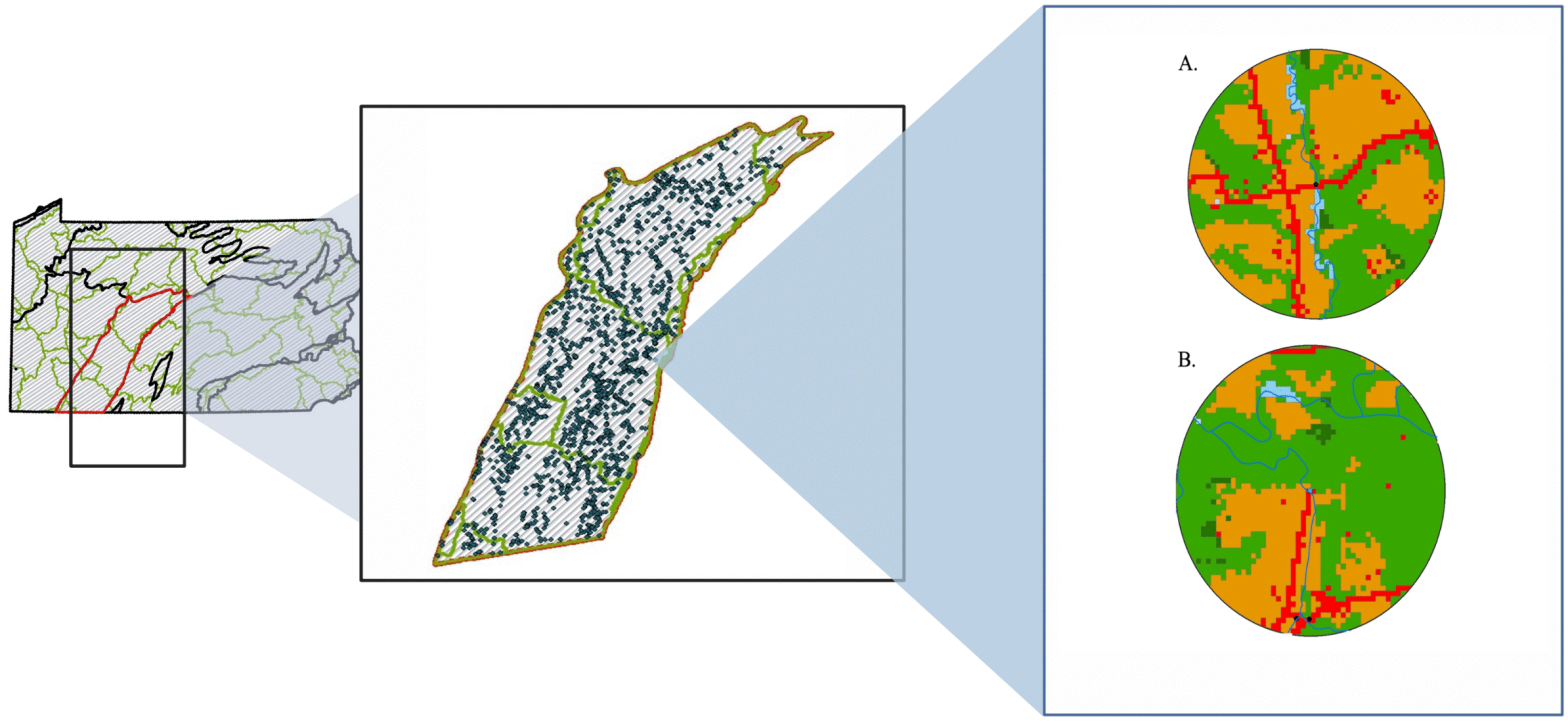
Study region and representative land sharing and land sparing configurations. From left to right. The first image shows level III ecoregions outlined in black (ecoregion 69 is out lined in red); within the ecoregions Hydrologic Unit Code (HUC) 8 subbasins are outlined in green. The second, middle image shows ecoregion 69 with its respective HUC 8 subbasins and blue dots which represent access point (i.e., bridges). The last panel shows a set of paired sampling sites (i.e., streams) flowing through watersheds that represent land sharing (A) and land sparing (B) configurations for agriculture (orange habitat) and forests (green habitat). Red areas indicate roads and other urban structures such as farmhouses and animal enclosures. All spatial data used in this figure were obtained from publicly available sources and were open for use without restriction.

To characterize spatial configuration of land use, land cover data from the National Land Cover Database [38] were reclassified into seven categories: water, developed, barren, forest, shrubland/herbaceous, agriculture, and wetlands. These data were integrated into ArcGIS 10.8.1, and overlaid onto geodata for ecoregions 67, 69, and 70, HUC 8 subbasins, river/stream access points (i.e., bridges), and land cover data (Fig 1).

To identify stream pairs within HUC 8 subbasins—one in a land sparing configuration and one in a land sharing configuration—public access points (i.e., bridges) within 50-m of 3rd or 4th order streams were identified visually using ArcGIS. Only streams passing through agricultural and natural land were considered; thus, we excluded urban sites and those flowing through wetlands or barren land. After identifying public access points, a 2.0-km^2^ buffer circle (800-m radius) was drawn around access points and used to quantify the spatial configuration of habitats in the surrounding landscape. The buffer circle provided a consistent spatial extent for comparing land use around chosen stream sites and captured land-use influences in all directions relative to the focal stream reach. Compared to irregular or linear buffer shapes, circular buffers reduce spatial bias introduced by stream orientation or landscape features and are commonly used in stream ecology to standardize the analysis of local land use and its effects on aquatic biodiversity [39–40]. The 2.0-km^2^ size of the buffer circle ensured we had a reach of sufficient length to sample four riffle habitats flowing through both agricultural and forested habitats. We focused on riffles because they are areas of high macroinvertebrate diversity in streams and are often the habitat of focus for biomonitoring [41].

Landscape spatial configuration within the circular buffer was quantified using Moran’s I, an index of spatial autocorrelation that measures the degree to which similar land cover/habitat types are clustered or dispersed across space. Values of Moran’s I range from −1 (perfect dispersion of land cover types; which is representative of land sharing) to +1 (perfect clustering of similar land use types, which is representative of land sparing), with values near zero indicating a random distribution of land use types. Moran’s I was selected because it is a widely utilized statistic in geospatial research for assessing spatial autocorrelation and has previously been used in LSLS studies [42–44]. Moran’s I was calculated for all bridge access points within each HUC 8 subbasin until a maximum of 40 sites were selected. The two most clustered (highest Moran’s I) and two most dispersed (lowest Moran’s I) sites were then chosen for sampling in each subbasin as described next. Substitutions of streams were made if access to a site was deemed unavailable during field sampling.

### Stream sampling

Four riffles were sampled in each stream reach, including two riffles located up to 400-m upstream of the access point and two riffles located up to 400-m downstream. Each riffle was at a minimum of 100m away from any other sampled riffle. Thus, the total length of stream reach sampled was approximately 800-m, corresponding to the radius of land use analyzed for spatial configuration. Each riffle in a stream was sampled for macroinvertebrate diversity using a 1m x 1m, 500µm mesh kick net. Kick net samples were placed in a 500µm mesh sieve, and large debris and vertebrates were removed. Samples were then stored in polyethylene plastic bottles and preserved with 70% ethanol for later enumeration and identification (see Macroinvertebrate diversity below). No state or federal permits were required for macroinvertebrate sampling at publicly accessible bridge crossings. Sampling was conducted from public access points and did not involve restricted waterways.

### Covariates

At each riffle, we measured a suite of covariates that have the potential to influence macroinvertebrate diversity, composition, and abundance. Doing so enabled us (a) to verify that covariates had been successfully standardized (i.e., held constant) among stream pairs by the study design, or alternatively, (b) to statistically control for any covariates that had not been standardized in the data analyses. Water velocity was measured at the center of each riffle at 50% of the total depth using a SonTek FlowTracker handheld acoustic Doppler velocimeter. Water temperature and conductivity were measured using a YSI Model 85 conductivity meter. Water pH was measured with an Oakton Premium 50 Series handheld meter. Canopy cover above the riffle was quantified using a spherical crown densitometer, and stream width was measured across the wetted perimeter using a measuring tape. Sediment size was characterized by collecting thirty rocks sampled at equidistant points along transects perpendicular to the riffle and measuring them with a Wildco Gravelometer that had sieve sizes ranging from 2 mm to 180 mm. Because the smallest measurable size class using a gravelometer is 2 mm, particles finer than this threshold (i.e., silt and clay) are not reliably quantified using this approach. Consequently, while our measurements characterize substrate particle size structure, they do not provide a precise estimate of the percent areal cover of deposited fine sediment (<2 mm) within riffles. Concentrations of nitrate (NO_3_^−^) and phosphate (PO_4_^3−^) were determined from filtered water samples collected in 50 mL centrifuge tubes using GFF microfiber filters (diameter: 47 mm, pore size: 0.5 µm) and frozen until analysis. Nitrate was measured using the nitrate reductase–NED method, a colorimetric assay involving reduction of nitrate to nitrite followed by reaction with N-(1-naphthyl)ethylenediamine dihydrochloride (NED) [45]. Phosphate concentrations were determined spectrophotometrically using a reagent mix containing ammonium molybdate, potassium antimony tartrate, and sulfuric acid [46–47]. Absorbance for both colorimetric assays was measured on a BioTek Synergy H1 plate reader.

Algal biofilm in each riffle was sampled by scraping 32 mm × 32 mm sections of three haphazardly chosen rocks from the middle of the riffle into a 50 ml centrifuge tube to quantify ash-free dry weight (AFDW) and chlorophyll-a concentration. To estimate AFDW, 20 ml of homogenized biofilm was filtered onto GFF filter paper using a syringe filter, dried, pre-weighed, and ashed in a muffle furnace at 600°C. Chlorophyll-a was quantified by filtering the remaining biofilm through a 25 mm syringe filter, placing the filter in 90% ethanol within a 15 ml centrifuge tube, and measuring the extract fluorometrically using a BioTek Synergy H1 microplate reader.

### Macroinvertebrate diversity

To characterize stream macroinvertebrate diversity, preserved samples from all four riffles within each stream were combined and poured into a stainless-steel autoclave tray that was divided into 48 squares (each 15.25 × 15.25 cm). The material was then homogenized across all squares to ensure an even distribution of organisms prior to subsampling. Squares were picked in random order and all invertebrates within a square were enumerated and identified using dissection and compound microscopes and dichotomous keys in [48–49]. We continued the process of picking squares at random and identifying individuals until a sampling accumulation curve (# invertebrate taxa discovered vs. # individual invertebrate organisms sampled) revealed no new taxa being identified for a minimum of 25 identifications. Sampling continued until taxa accumulation curves approached an asymptote (i.e., no new taxa were detected for at least 25 consecutive individuals), indicating that additional sampling was unlikely to substantially increase observed taxa richness. This approach provided confidence that sampling effort was sufficient to capture the vast majority of taxa present within each stream reach. All organisms were identified to the lowest taxonomic group possible, which was usually genus for insects, but family for other invertebrates like bivalves, gastropods, crustaceans, and annelids [48–49]. Across the 72 sampled stream reaches, a mean of 63.9 individuals was enumerated and identified per stream (median = 49.5, minimum = 7.0, maximum = 246.0).

### Statistical analysis

We began data analyses by calculating pairwise differences for all dependent and independent variables between paired stream sites. Each HUC 8 subbasin contained the two highest (most clustered) and two lowest (most dispersed) Moran’s I streams, giving the potential for 4 paired comparison per watershed. For each possible comparison, differences were computed by subtracting values from the more dispersed site (i.e., lower Moran’s I, representing land sharing) from those of the more clustered site (i.e., higher Moran’s I, representing land sparing). Specifically, differences were calculated between D1 and C1 (most dispersed vs. most clustered), D2 and C2 (second most dispersed vs. second most clustered), D2 and C1, and D1 and C2. The resulting dataset of differences among dispersed and clustered paired sites was then imported into the software program R for statistical analysis.

In R (version 4.4.3), we first tested whether potential covariates (i.e., potential explanatory variables) differed among stream pairs using parametric (paired t-tests) or non-parametric (Wilcoxon signed-rank) tests, depending on the distribution of the data. We then fit generalized linear models (GLMs) of the form: $Y=\;\beta_{\text{0}}+\beta_{i}*{Cov}_{i}$, where Y represents the difference in macroinvertebrate taxa richness, evenness, or abundance between paired streams, β₀ is the overall mean difference among all 72 paired comparisons, and βᵢ represents the effect of any covariate i that had not been not held constant by the paired study design (i.e., showed a significant difference among pairs).

To further evaluate whether land use configurations differed in overall environmental context, we conducted a principal components analysis (PCA) on standardized environmental covariates to reduce dimensionality and account for collinearity among variables. The resulting principal component scores were used to characterize dominant environmental gradients across sites. Differences in multivariate environmental space between land sharing and land sparing sites were then tested using permutational multivariate analysis of variance (PERMANOVA) on Euclidean distances of the standardized environmental data.

Non-metric multidimensional scaling (NMDS) was then used to assess and visualize differences in macroinvertebrate community composition between land sharing and land sparing stream sites. The analysis was conducted in R version 4.4.3 using the vegan package, where taxa abundance data were organized into a site-by-taxon matrix with zeros removed from the dataset. Taxa abundances were Hellinger-transformed prior to analysis to reduce the influence of hyperabundant taxa while retaining information from rare taxa. A Bray-Curtis dissimilarity matrix was calculated from the transformed community matrix, and NMDS was applied to reduce the multivariate community data into two-dimensional ordination space. To statistically test for differences in community composition between land use types, a permutational multivariate analysis of variance (PERMANOVA) was performed on the Bray–Curtis dissimilarity matrix using the adonis2 function in the vegan package, with permutations restricted within paired sites to account for the matched study design.

Lastly, macroinvertebrates were classified into one of six functional feeding groups based on published literature and online resources [49–51]. To evaluate differences in functional group abundance, we used linear mixed-effects models with conductivity and phosphate (P) concentration as fixed effects and subbasin as a random effect. Conductivity and phosphate were included as covariates because they differed between land use configurations (i.e., differed significantly among pairs) and were not held constant by the paired design.

## Results

The paired stream study design was successful at selecting streams that ran through different land use configurations (see Moran’s I, Table 1) while simultaneously holding most of the covariates constant (Table 1). Indeed, Moran’s I differed significantly between land sharing and sparing streams (t = 23.17, P < 0.001) with values for sharing streams that were significantly negative, and values for sparing streams that were positive and 1.4x greater. Of the 12 covariates that were measured, all physical habitat characteristics (sediment size, canopy cover, stream width, depth, and velocity) were the same among stream pairs, and most of the water quality parameters (water pH, temperature, nitrate-N, ash-free dry weight AFDW, and chlorophyll concentration) were also held constant. Because these covariates did not differ among stream pairs, they cannot be responsible for any differences in macroinvertebrate diversity found among pairs. Of the covariates measured, only two water quality parameters—phosphate concentration and conductivity—showed marginal differences among stream pairs and were not fully controlled by the paired study design (Table 1). Therefore, we statistically controlled for these two covariates in subsequent analyses.

**Table 1:** Covariate measurements for land sharing and land sparing configurations.

Covariate	Sharing-Avg (±SE)	Sparing-Avg (± SE)	Test	Statistic	P
Moran’s I	−0.159 ± 0.009	0.219 ± 0.013	t-test	t = 23.17	<.001^*^
P (µM)	5.69 ± 1.21	6.67 ± 1.00	Wilcoxon	W = 814.0	0.06
Conductivity (µS/cm)	360.55 ± 29.12	278.27 ± 19.06	Wilcoxon	W = 480.0	0.06
Sediment (mm)	91.01 ± 6.05	85.59 ± 6.03	t-test	t = −0.63	0.52
AFDW (g/L)	0.061 ± 0.005	0.044 ± 0.011	Wilcoxon	W = 652.5	0.96
Chlorophyll (mg/mL)	0.395 ± 0.050	0.357 ± 0.060	Wilcoxon	W = 562.0	0.34
N (µM)	63.74 ± 8.85	57.33 ± 7.26	Wilcoxon	W = 635.5	0.89
Canopy Cover (%)	40.29 ± 3.12	40.32 ± 3.62	t-test	t = 0.03	0.98
pH	7.69 ± 0.08	7.83 ± 0.09	t-test	t = 1.064	0.29
Temperature (°F)	22.77 ± 0.47	22.94 ± 0.48	t-test	t = 0.25	0.80
Stream Width (inches)	191.85 ± 16.75	176.25 ± 15.55	Wilcoxon	W = 592.5	0.54
Stream Depth (inches)	5.78 ± 0.82	4.56 ± 0.27	Wilcoxon	W = 589.5	0.51
Stream Velocity (m/s)	3.04 ± 0.43	2.48 ± 0.39	t-test	t = −0.94	0.34

Summary of environmental covariates between sharing and sparing sites. Values are presented as means ± standard error. Statistical comparisons were conducted on untransformed data. Parametric variables were analyzed using t-tests, while non-parametric variables were assessed using Wilcoxon rank-sum tests. Test statistics and corresponding p-values are provided. The sample size (N) for each covariate was 36, except for stream velocity (sharing, N = 35; sparing, N = 34). Abbreviations: AFDW = ash-free dry weight; P = phosphorus concentration (µM); N = nitrogen concentration (µM); µS/cm = microsiemens per centimeter; m/s = meters per second; mg/mL = milligrams per milliliter; µM = micromoles per liter; SE = standard error.

To further evaluate whether environmental conditions differed collectively between land use configurations, we conducted PCA on standardized environmental variables and tested for multivariate differences using PERMANOVA. Although combining covariates into multivariate environmental space increased the apparent separation along primary axes, land sharing and land sparing streams exhibited substantial overlap and did not differ significantly in overall environmental context (PERMANOVA: R² = 0.016, P = 0.17). Thus, even when considered collectively, environmental conditions did not differ between land use configurations.

**Fig 2:**
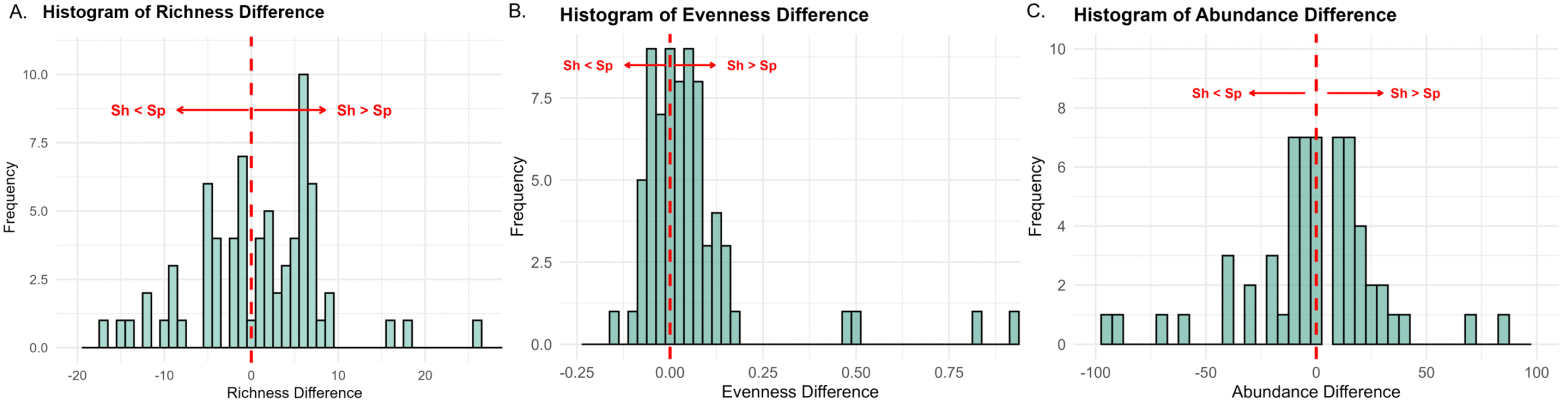
Histograms for (a) Taxa richness, (b) taxa evenness, and (c) total abundance. Histograms showing the distribution of differences in macroinvertebrate community metrics between land sharing and sparing stream reaches. (a) Taxa richness, (b) taxa evenness, and (c) total abundance. The red vertical line represents zero, indicating no difference between configurations for paired streams; values to the left of the line indicate greater values in sharing sites, and values to the right indicate greater values in sparing sites. Abbreviations: Sh = land sharing, Sp = land sparing.

Two of the paired differences between land sparing vs. sharing streams proved to be statistical outliers that were at 2.5 standard deviations or more beyond the mean difference. We decided to remove these outliers from analyses to minimize the influence of extreme values, though we still show them in Fig 2a–2c for reference. After outliers were removed, we found no significant differences in macroinvertebrate taxa richness, evenness, or total abundance between land sharing and sparing stream reaches (Fig 2a–2c). There was considerable variation in differences for richness, evenness, and abundance among paired stream sites, but the distribution of these differences centered around a mean of zero. After controlling for conductivity and phosphate (P) concentrations, richness did not differ significantly among stream pairs (t = 0.41, p = 0.68), though richness did exhibit a negative correlation with P (t = –2.46, p = 0.02; S1–S1.1 Table). Evenness did not differ among stream pairs and neither conductivity nor P was related to evenness (t = 1.37, p = 0.19; S2–S2.1 Table). Total abundance did not differ between stream pairs (t = 0.61, p = 0.55), although abundance was negatively related to P differences among stream pairs (t = –1.72, p = 0.09; S3–S3.1 Table).

**Fig 3:**
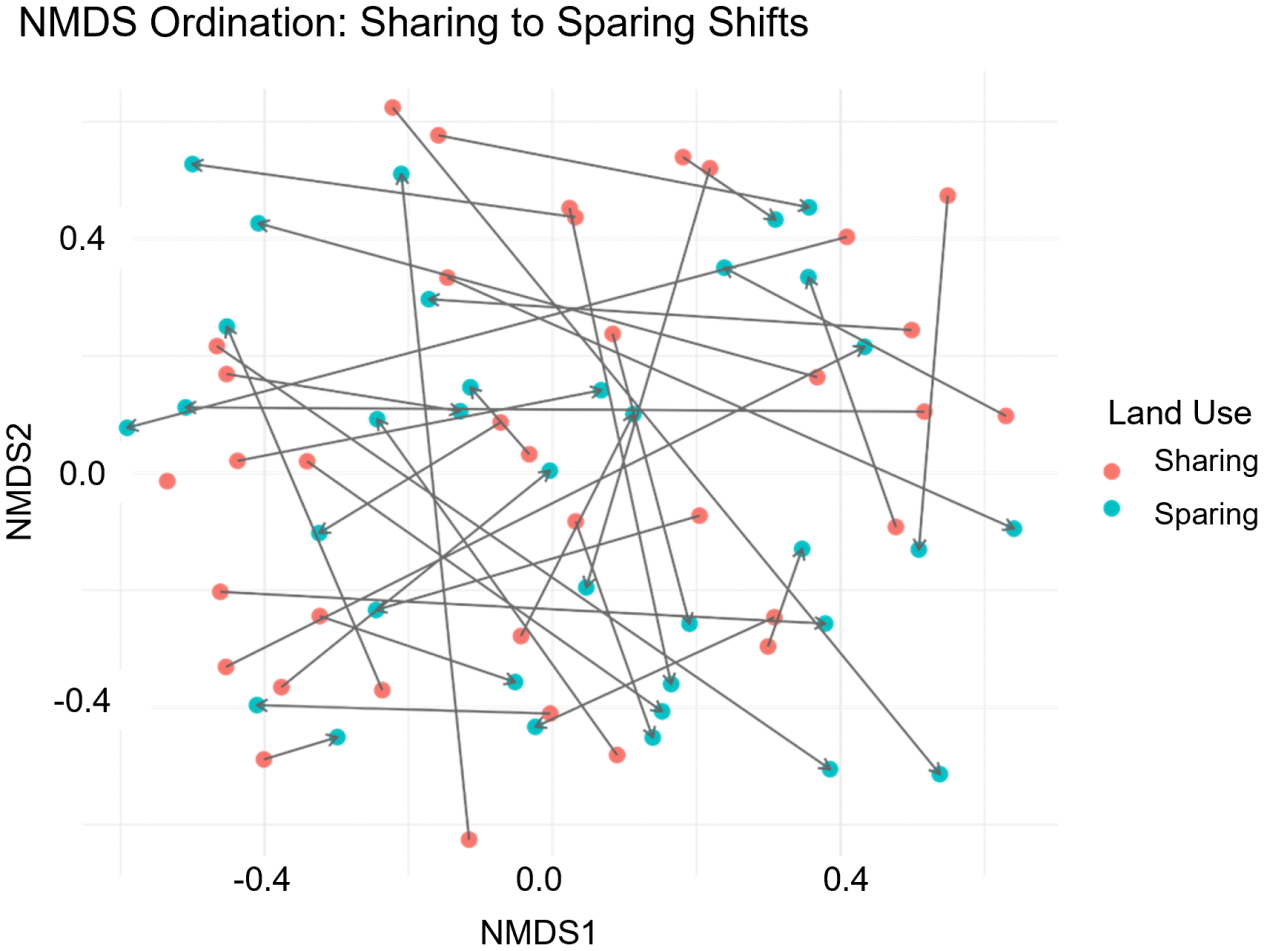
Community composition NMDS. Non-metric multidimensional scaling (NMDS) ordination of macroinvertebrate community composition in land sharing and sparing stream reaches. Each point represents a stream reach, and proximity between points reflects similarity in community composition. No distinct clustering between sharing and sparing sites was observed. Stress value and PERMANOVA results are provided in the text.

We further found no difference in macroinvertebrate community composition between land sharing and sparing stream pairs (F = 0.015, p = 0.21). If consistent differences had existed, we would expect paired streams to diverge along NMDS axes. However, as evident in Fig 3, NMDS revealed no clear clustering or directional separation between the two stream types (Fig 3).

**Fig 4:**
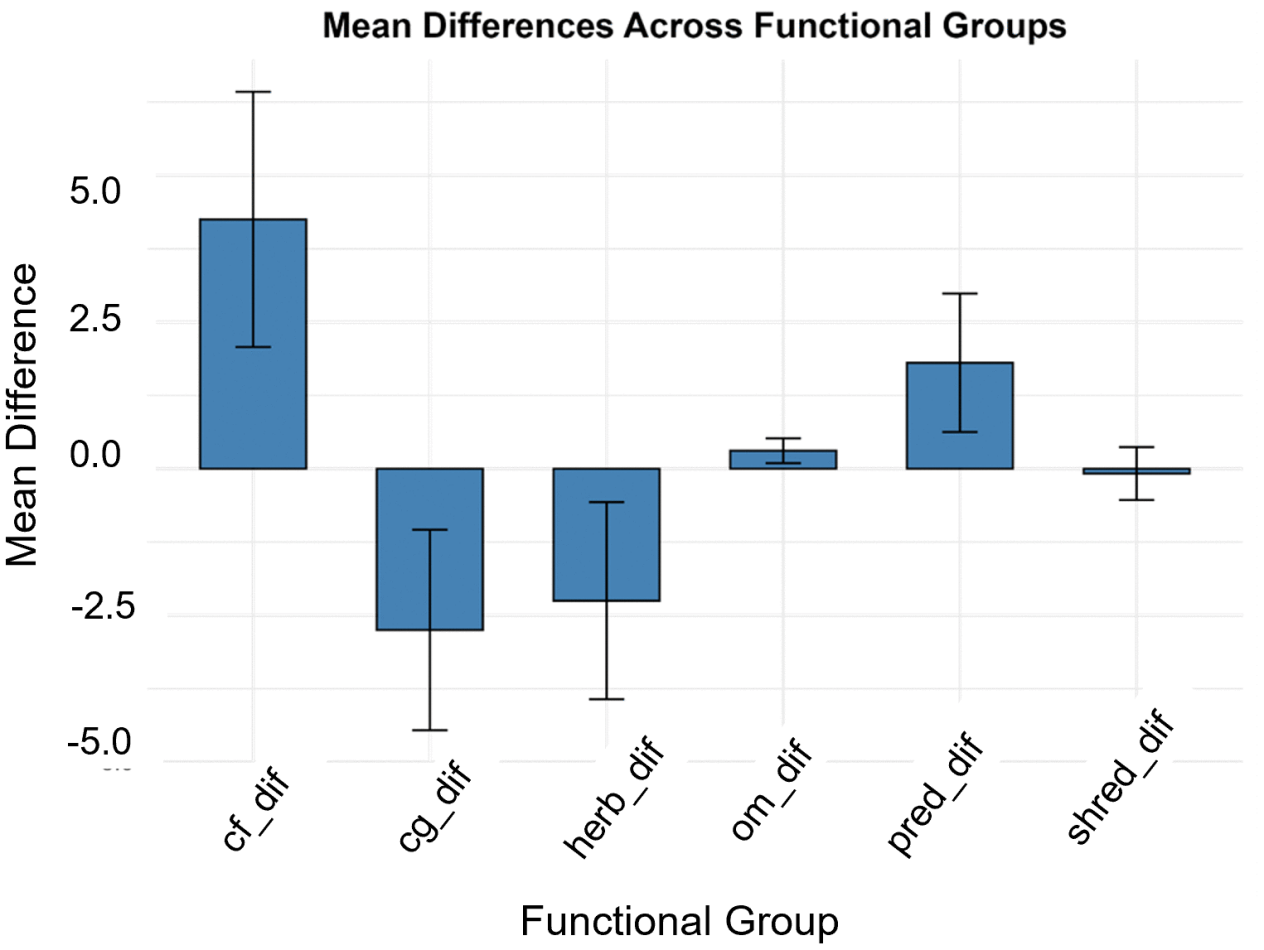
FFG abundance. Differences in functional feeding group (FFG) composition between land sharing and sparing stream reaches. Positive values indicate greater abundance for sharing sites and negative indicates greater abundance in sparing sites. Abbreviations: CF = collector-filterers, CG = collector-gatherers, Herb = herbivores, OM = omnivores, Pred = predators, Shred = shredders.

We did find minor differences in Functional Feeding Group (FFG) composition between land sharing and sparing stream reaches (Fig 4). Of the six FFGs, only collector-filterers differed significantly between land sharing and land sparing streams (t = 2.55, p = 0.02; S4.1 Table), though the effect was small, averaging just four more individuals in land sharing sites. No other groups differed significantly by configuration. However, differences in shredder abundance were significantly correlated with conductivity between stream pairs (t = –2.27, p = 0.03; S4.2 Table).

## Discussion

Land sharing and land sparing have been proposed as two alternative strategies for balancing biodiversity conservation with increasing global food demand—an increasingly urgent challenge as governments strive to meet ambitious conservation targets such as the 30% land/water conservation by 2030 goals of the Global Biodiversity Framework [52–53]. While terrestrial studies have produced mixed evidence supporting both land sparing and land sharing approaches, their implications for aquatic ecosystems embedded within these landscapes remain largely unexplored.

Despite the growing body of terrestrial research on land sharing and sparing, our study is one of if not the first to apply this framework to freshwater ecosystems [12,23]. Streams and rivers are closely tied to surrounding land use and often flow through agricultural landscapes where conservation trade-offs are pressing [54]. Evaluating how spatial land use configurations influence aquatic biodiversity is critical for informing cross-realm conservation strategies, particularly given that land use planning often prioritizes terrestrial outcomes over freshwater ones [55]. Understanding whether land sharing or sparing principles hold relevance for aquatic biodiversity can help refine the scope and application of the LSLS framework beyond terrestrial contexts.

Our study found no evidence that land use configuration *per se*, whether agricultural land is integrated with or spatially separated from more natural forested areas, affects macroinvertebrate diversity in stream ecosystems. Richness, evenness, total abundance, and overall community composition did not differ significantly between stream reaches embedded in land sharing versus land sparing landscapes. Our results challenge a key assumption underlying much of the terrestrial literature: that the spatial arrangement of land use plays a central role in shaping biodiversity outcomes [56–58]. In contrast to terrestrial systems, where landscape configuration can influence species persistence through effects on habitat fragmentation and dispersal corridors, aquatic ecosystems may be structured by different spatial and ecological forces [59–60].

The lack of a discernable effect of spatial habitat configuration aligns with a growing body of freshwater research suggesting that linear connectivity, hydrologic processes, and upstream-downstream linkages may override any minor influence of land use patterns immediately surrounding stream reaches [61–62]. In lotic systems, directional flow can transport materials and organisms downstream, potentially making upstream conditions more influential than adjacent land use. Future studies should examine how upstream land sharing and sparing configurations affect downstream ecological responses. Similarly, hydrologic processes like flow variability and groundwater exchange also shape invertebrate communities and may override local land use effects [63]. The absence of an effect from land use configuration implies that conservation frameworks developed for terrestrial environments may not translate directly to stream systems, and it underscores the need for freshwater-specific strategies that consider the unique spatial constraints and dispersal pathways of aquatic organisms.

While our study found no effect of land sharing or sparing configurations on aquatic macroinvertebrate diversity, numerous terrestrial studies have demonstrated that landscape configuration does influence biodiversity. For instance, land sparing configurations have been shown to enhanced dung beetle diversity by reducing habitat fragmentation and minimizing edge effects [15]. Conversely, other studies have found that land sharing approaches can promote terrestrial insect diversity by facilitating movement and dispersal across heterogeneous landscapes, thereby increasing resilience to local disturbances [18,22]. Moreover, studies have shown that different groups of organisms may respond variably to land sharing and land sparing strategies, indicating that no single approach is universally effective for conserving biodiversity across all groups [64]. Combined with our results, these contrasting findings from terrestrial LSLS studies suggest that land sharing and land sparing strategies do not have uniform effects across all ecosystems or taxonomic groups.

The absence of a configuration effect should, in no way, be taken as evidence that macroinvertebrate communities in streams are unaffected by agricultural land use. Numerous studies have already documented widespread impacts of increasing agricultural intensity on macroinvertebrate diversity. For instance, pesticide contamination from agricultural runoff can shift community composition toward more pollution-tolerant taxa — a pattern that is well established in the literature [65–67]. Additionally, intensified agriculture is associated with increased erosion and stream sedimentation, both of which negatively affect macroinvertebrate diversity [68]. Consistent with prior work, richness across all streams in our study was negatively correlated with conductivity, which is a common proxy for agricultural intensity (t = –1.92, P = 0.06) [69]. However, our paired stream design was specifically used to minimize variation in physical and chemical conditions between site pairs. Once the confounding influence of these physicochemical conditions was controlled for, we found no evidence that land use configuration per se, when isolated from other factors, affected macroinvertebrate diversity.

Although our study found no effect of spatial habitat autocorrelation (as measured by Moran’s I) on diversity of aquatic macroinvertebrates, we acknowledge that many other studies have shown that other spatial patterns in landscapes do, in fact, influence species diversity. For example, substantial research has demonstrated that factors like spatial habitat heterogeneity and patch proportions can shape biodiversity [70–72]. Therefore, our findings should not be interpreted as evidence that spatial patterns are unimportant to stream macroinvertebrates, but rather that spatial configuration, as quantified by a specific metric of autocorrelation, did not significantly relate to biodiversity at this scale.

The spatial scale of our study (2 km² extent) was selected partly for practical reasons (i.e., constraints on sampling distances). Even so, we believe the spatial extent of our study is likely to reflect a biologically relevant scale for many stream macroinvertebrates whose adult stages often disperse, mate, and oviposit within in close proximity to their emergence sites [73–74]. Nevertheless, it remains possible that analyses conducted at broader spatial scales could yield different results. In fact, recent research found that various metrics of landscape patterns (i.e., spatial configuration of land cover classes and percent land cover) explained more variation in benthic macroinvertebrate communities at the extent of entire watersheds than when measured at finer spatial extents [75]. Given this, we cannot eliminate the possibility that future studies might find that stream macroinvertebrates are influenced by spatial autocorrelation of habitats at scales larger than we were able to study.

Our findings call for a critical re-evaluation of how land sharing and sparing frameworks are applied across ecological realms. While terrestrial studies have often emphasized the importance of spatial configuration in shaping biodiversity, our results suggest that this assumption may not hold in stream ecosystems. We found no evidence that the arrangement of agricultural and forested land, whether integrated or spatially separated, affected macroinvertebrate diversity, abundance, or community composition at the stream-reach scale. Adapting these frameworks for aquatic systems will require new approaches that reflect the unique ecological processes governing rivers and streams.

## Supporting information

S1 Table Linear Mixed Model (LMM) estimates of covariate effects on taxa richness difference. These tables present linear regression results examining the relationships between differences in conductivity (Cond) and phosphorus (P) and differences in taxa richness between paired land-sharing and land-sparing stream reaches. Results are shown with (Table A) and without (Table B) outliers. SE = standard error.
(DOCX)

S2 Table Linear Mixed Model (LMM) estimates of covariate effects on evenness difference. These tables present linear regression results testing whether differences in conductivity (Cond) and phosphorus (P) between paired land-sharing and land-sparing stream reaches were associated with differences in taxa evenness between the same paired reaches. Results are shown with (Table A) and without (Table B) outliers. Asterisks indicate statistically significant regression coefficients (p < 0.05). SE = standard error.
(DOCX)

S3 Table Linear Mixed Model (LMM) estimates of covariate effects on abundance difference. These tables present linear regression results examining the relationships between differences in conductivity (Cond) and phosphorus (P) and differences in taxa abundance between paired land-sharing and land-sparing stream reaches. Results are shown with (Table A) and without (Table B) outliers. SE = standard error.
(DOCX)

S4 Table Linear Mixed Model (LMM) estimates of covariate effects on FFG differences. These tables present linear regression results examining the relationships between differences in conductivity (Cond) and phosphorus (P) and differences in functional feeding group (FFG) abundance between paired land-sharing and land-sparing stream reaches for collector-filterers (Table A), collector-gatherers (Table B), herbivores (Table C), omnivores (Table D), predators (table E), and shredders (Table F). Asterisks indicate statistically significant regression coefficients (p < 0.05). SE = standard error. Abbreviations: CF = collector-filterers, CG = collector-gatherers, Herb = herbivores, OM = omnivores, Pred = predators, Shred = shredders.
(DOCX)

S5 Table Results of the principal components analysis (PCA) conducted on standardized environmental variables measured across sampled stream reaches. Table A presents the standard deviation, proportion of variance explained, and cumulative proportion of variance explained for each principal component. Table B presents PCA loadings for environmental variables included in the analysis, where larger absolute loading values indicate stronger contributions to a principal component axis. Positive and negative values indicate the direction of variable associations with component axes. Abbreviations: PC = Principal Component; AFDW = ash-free dry weight; N = nitrogen concentration; P = phosphorus concentration; Chyl_Con = chlorophyll concentration; V = water velocity; Cond = conductivity; Temp = water temperature; Can_cover = canopy cover.
(DOCX)
