# Supplementary material for: Land sparing or sharing: A paired stream study to evaluate the relationship between land use configuration and macroinvertebrate diversity in streams
Source: PLoS One. 2026 Aug 12;21(8):e0354515. doi: 10.1371/journal.pone.0354515 (PMC13465839; doi:10.1371/journal.pone.0354515)
Supplement: Supplementary file 1 [file pone.0354515.s001.docx]

**S1Table. Linear Mixed Model (LMM) Estimates of Covariate Effects on Richness Difference.** These tables present linear regression results examining the relationships between differences in conductivity (Cond) and phosphorus (P) and differences in taxa richness between paired land-sharing and land-sparing stream reaches. Results are shown with (Table A) and without (Table B) outliers. SE = standard error.

Table A

| **Variable** | **Estimate ± SE** | **T-Statistic** | **P-Value** |
| --- | --- | --- | --- |
| Richness_dif (β_0_) | 1.08 ± 1.23 | 0.87 | 0.39 |
| Cond_dif (β_1_) | -0.005 ± 0.004 | -1.17 | 0.24 |
| P_dif (β_2_) | -0.26 ± 0.14 | -1.87 | 0.07 |

Table B outliers removed

| **Variable** | **Estimate ± SE** | **T-Statistic** | **P-Value** |
| --- | --- | --- | --- |
| Richness_dif (β_0_) | 0.48 ± 1.16 | 0.41 | 0.68 |
| Cond_dif (β_1_) | -0.003 ± 0.004 | -0.64 | 0.52 |
| P_dif (β_2_) | -0.31 ± 0.13 | -2.46 | 0.02 |
