# Supplementary material for: Land sparing or sharing: A paired stream study to evaluate the relationship between land use configuration and macroinvertebrate diversity in streams
Source: PLoS One. 2026 Aug 12;21(8):e0354515. doi: 10.1371/journal.pone.0354515 (PMC13465839; doi:10.1371/journal.pone.0354515)
Supplement: Supplementary file 2 [file pone.0354515.s002.docx]

**S2 Table. Linear Mixed Model (LMM) Estimates of Covariate Effects on Evenness Difference.** These tables present linear regression results testing whether differences in conductivity (Cond) and phosphorus (P) between paired land-sharing and land-sparing stream reaches were associated with differences in taxa evenness between the same paired reaches. Results are shown with (Table A) and without (Table B) outliers. Asterisks indicate statistically significant regression coefficients (p < 0.05). SE = standard error.

Table A.

| **Variable** | **Estimate ± SE** | **T-Statistic** | **P-Value** |
| --- | --- | --- | --- |
| Evenness_dif (β_0_) | 0.67 ± 0.03 | 2.21 | **0.04^*^** |
| Cond_dif (β_1_) | -0.0002 ± 0.0001 | -1.49 | 0.14 |
| P_dif (β_2_) | -0.26 ± 0.14 | -0.81 | 0.42 |

Table B outlier removed

| **Variable** | **Estimate ± SE** | **T-Statistic** | **P-Value** |
| --- | --- | --- | --- |
| Evenness_dif (β_0_) | 0.02 ± 0.015 | 1.37 | 0.19 |
| Cond_dif (β_1_) | 0.00001 ± 0.00003 | 0.22 | 0.83 |
| P_dif (β_2_) | 0.0001 ± 0.001 | 0.05 | 0.96 |
