# Supplementary material for: Land sparing or sharing: A paired stream study to evaluate the relationship between land use configuration and macroinvertebrate diversity in streams
Source: PLoS One. 2026 Aug 12;21(8):e0354515. doi: 10.1371/journal.pone.0354515 (PMC13465839; doi:10.1371/journal.pone.0354515)
Supplement: Supplementary file 3 [file pone.0354515.s003.docx]

**S3 Table. Linear Mixed Model (LMM) Estimates of Covariate Effects on Abundance Difference.** These tables present linear regression results examining the relationships between differences in conductivity (Cond) and phosphorus (P) and differences in taxa abundance between paired land-sharing and land-sparing stream reaches. Results are shown with (Table A) and without (Table B) outliers. SE = standard error.

Table A.

| **Variable** | **Estimate ± SE** | **T-Statistic** | **P-Value** |
| --- | --- | --- | --- |
| Abundance_dif (β_0_) | 2.41 **±** 3.96 | 0.61 | 0.55 |
| Cond_dif (β_1_) | -0.03 **±** 0.02 | -1.32 | 0.19 |
| P_dif (β_2_) | -0.96 **±** 0.56 | -1.72 | 0.09 |

Table B (outliers removed)

| **Variable** | **Estimate ± SE** | **T-Statistic** | **P-Value** |
| --- | --- | --- | --- |
| Abundance_dif (β_0_) | 2.35 **±** 3.41 | 0.69 | 0.49 |
| Cond_dif (β_1_) | 0.002 **±** 0.011 | 0.18 | 0.86 |
| P_dif (β_2_) | 0.31 **±** 0.36 | 0.85 | 0.39 |
