# Supplementary material for: Land sparing or sharing: A paired stream study to evaluate the relationship between land use configuration and macroinvertebrate diversity in streams
Source: PLoS One. 2026 Aug 12;21(8):e0354515. doi: 10.1371/journal.pone.0354515 (PMC13465839; doi:10.1371/journal.pone.0354515)
Supplement: Supplementary file 5 [file pone.0354515.s005.docx]

**S5 Table. Results of the principal components analysis (PCA) conducted on standardized environmental variables measured across sampled stream reaches.** Table A presents the standard deviation, proportion of variance explained, and cumulative proportion of variance explained for each principal component. Table B presents PCA loadings for environmental variables included in the analysis, where larger absolute loading values indicate stronger contributions to a principal component axis. Positive and negative values indicate the direction of variable associations with component axes. Abbreviations: PC = Principal Component; AFDW = ash-free dry weight; N = nitrogen concentration; P = phosphorus concentration; Chyl_Con = chlorophyll concentration; V = water velocity; Cond = conductivity; Temp = water temperature; Can_cover = canopy cover.

Table A Variance Explained by Principal Components

| **PC** | **Standard deviation** | **Variance explained (%)** | **Cumulative variance (%)** |
| --- | --- | --- | --- |
| PC1 | 1.55 | 20.0 | 20.0 |
| PC2 | 1.26 | 13.20 | 33.20 |
| PC3 | 1.22 | 12.40 | 45.70 |
| PC4 | 1.10 | 10.10 | 55.80 |
| PC5 | 1.01 | 8.40 | 64.20 |
| PC6 | 0.97 | 7.90 | 72.10 |

Table B. PCA Loadings for Environmental Variables

| **Variable** | **PC1** | **PC2** | **PC3** |
| --- | --- | --- | --- |
| Sediment_size | 0.29 | -0.03 | -0.39 |
| Can_cover | 0.15 | -0.46 | -0.19 |
| pH | 0.29 | 0.12 | -0.17 |
| Cond | 0.08 | -0.26 | 0.16 |
| Temp | -0.15 | -0.07 | -0.62 |
| AFDW | 0.26 | 0.19 | 0.45 |
| N | 0.45 | 0.17 | -0.03 |
| P | 0.21 | 0.48 | 0.02 |
| Chyl_Con | 0.42 | 0.26 | -0.24 |
| Width | 0.38 | -0.25 | -0.05 |
| Depth | 0.33 | -0.39 | 0.32 |
| V | 0.19 | -0.33 | -0.02 |
