# Supplementary material for: Land sparing or sharing: A paired stream study to evaluate the relationship between land use configuration and macroinvertebrate diversity in streams
Source: PLoS One. 2026 Aug 12;21(8):e0354515. doi: 10.1371/journal.pone.0354515 (PMC13465839; doi:10.1371/journal.pone.0354515)
Supplement: Supplementary file 12 [file pone.0354515.s013.docx]

April 18^th^, 2026

Re: Revision of manuscript PONE-D-25-45509

Dear Dr. Marina Vilencia,

Thank you again for the opportunity to revise our manuscript PONE-D-25-45509 and for the constructive feedback provided by the reviewer. We have addressed the minor comments raised by the reviewer. Additionally, we have reviewed the reference list to assure that none of the works have been retracted.

Following this letter, we provide a point-by-point response explaining how we have addressed each reviewer comment. If you have any questions, concerns, or further requests for revision, we would be happy to address them. We look forward to your decision on this manuscript.

Sincerely,

Sugjit S. Padda, Ph.D.

Bradley J. Cardinale, Professor

**Response to Reviewer #1**

*We want to thank the reviewer for their insightful comments. They were very helpful and improved the manuscript. Please note the reviewer’s comments were not originally numbered; but for clarity and ease of reference, we have numbered them below and respond to each comment in turn.*

**Comment #1:** At L278 conductivity and phosphate concentrations are mentioned as covariates, presumably for the same logic described at L255. Making it explicitly clear at either instance of why conductivity and phosphate concentrations are used as covariates would help avoid any confusion for the reader. I expect that the procedure explained at L240-248 helps explain why Moran’s I was not included as a covariate in the GLMs despite differing between land use strategies.

*We have revised the Methods to explicitly clarify the rationale for including conductivity and phosphate as covariates, ensuring consistency with the earlier explanation and improving clarity for the reader (lines 279-281).*

**Comment #2:** The results of the PERMANOVA model testing macroinvertebrate species composition are duplicated at L331 and L337. The sentence at L335 is redundant here and could be moved/merged with the data analysis methods.

*We have removed the duplicate PERMANOVA results and revised the text to eliminate the redundancy (lines 333-337).*
