# Supplementary material for: Land sparing or sharing: A paired stream study to evaluate the relationship between land use configuration and macroinvertebrate diversity in streams
Source: PLoS One. 2026 Aug 12;21(8):e0354515. doi: 10.1371/journal.pone.0354515 (PMC13465839; doi:10.1371/journal.pone.0354515)
Supplement: Supplementary file 13 [file pone.0354515.s007.docx]

February 19^th^, 2026

Re: Revision of manuscript PONE-D-25-45509

Dear Dr. Marina Vilencia,

Thank you for the opportunity to revise our manuscript PONE-D-25-45509 and for the constructive feedback provided by the reviewer. We have given each comment serious consideration and have revised the manuscript accordingly. We believe these changes have improved the rigor of the study.

Following this letter, we provide a point-by-point response explaining how we have addressed each editorial and reviewer comment. If you have any questions, concerns, or further requests for revision, we would be happy to address them. We look forward to your decision on this manuscript.

Sincerely,

Sugjit S. Padda, Ph.D. Candidate

Bradley J. Cardinale, Professor

**Response to Academic Editor:**

**Comment #1:** Please ensure that your manuscript meets PLOS ONE's style requirements, including those for file naming.

*We have carefully revised the manuscript to ensure full compliance with PLOS ONE style requirements, including formatting and file naming conventions.*

**Comment #2:** In your Methods section, please provide additional information regarding the permits you obtained for the work. Please ensure you have included the full name of the authority that approved the field site access and, if no permits were required, a brief statement explaining why.

*We did not require state or federal permits for macroinvertebrate sampling because all collections were conducted at publicly accessible bridge crossings from public rights-of-way. Sampling did not involve restricted waterways or access to private property. We have clarified this in the Methods section to explicitly state that no permits were required and to identify the nature of site access (lines 186-188).*

**Comment #3:** Please note that your Data Availability Statement is currently missing [the repository name and/or the DOI/accession number of each dataset OR a direct link to access each database]. If your manuscript is accepted for publication, you will be asked to provide these details on a very short timeline. We therefore suggest that you provide this information now, though we will not hold up the peer review process if you are unable.

*All data and associated R code has been submitted to Dryad. The DOI identifier associated with the manuscript data is (*[*https://doi.org/10.5061/dryad.b8gtht7t0*](https://doi.org/10.5061/dryad.b8gtht7t0)*, and can be accessed temporarily through this link:*  [*http://datadryad.org/share/LINK_NOT_FOR_PUBLICATION/MtSAUGD-GII7qDemzlz0rD3_wi_04Q0yteFEXgSKzOY*](https://nam10.safelinks.protection.outlook.com/?url=http%3A%2F%2Fh52wvrds.r.us-west-2.awstrack.me%2FL0%2Fhttp%3A%252F%252Fdatadryad.org%252Fshare%252FLINK_NOT_FOR_PUBLICATION%252FMtSAUGD-GII7qDemzlz0rD3_wi_04Q0yteFEXgSKzOY%2F1%2F0101019c8bb91433-857ce607-6ee6-48bf-b3b6-147a04c3874f-000000%2F0da3u9K-iRwYUInUvvkzdVWs1Ps%3D467&data=05%7C02%7Csks6950%40psu.edu%7C3dece68993df4a11b2f508de7307e618%7C7cf48d453ddb4389a9c1c115526eb52e%7C0%7C0%7C639074674966131033%7CUnknown%7CTWFpbGZsb3d8eyJFbXB0eU1hcGkiOnRydWUsIlYiOiIwLjAuMDAwMCIsIlAiOiJXaW4zMiIsIkFOIjoiTWFpbCIsIldUIjoyfQ%3D%3D%7C0%7C%7C%7C&sdata=OeUI%2FVRd24WeWhWK26v16f5uJBGONUtn1aOLCzcQ0Nw%3D&reserved=0)*). The data will remain private for the peer review process and will be made public available after submission. The data availability statement can be found after the abstract in the manuscript (lines 47-50).*

**Comment #4:** We note that Figure 1 in your submission contain [map/satellite] images which may be copyrighted. All PLOS content is published under the Creative Commons Attribution License (CC BY 4.0), which means that the manuscript, images, and Supporting Information files will be freely available online, and any third party is permitted to access, download, copy, distribute, and use these materials in any way, even commercially, with proper attribution. For these reasons, we cannot publish previously copyrighted maps or satellite images created using proprietary data, such as Google software (Google Maps, Street View, and Earth).

*Figure 1 was created entirely using publicly available U.S. federal government geospatial datasets (EPA Level III Ecoregions, USGS HUC-8 subbasins, NHDPlus stream networks, NLCD land cover, and Pennsylvania Spatial Data Access). No proprietary basemaps (e.g., Google Maps, ESRI World Imagery, or other copyrighted satellite imagery) were used. All datasets are in the public domain and compatible with publication under a CC BY 4.0 license. The EPA states, “Unless otherwise specified, geospatial data produced by the EPA is by default in the public domain and is not subject to domestic copyright protection under 17 U.S.C. § 105.” (*[*EPA Disclaimers | US EPA*](https://www.epa.gov/web-policies-and-procedures/epa-disclaimers)*). The USGS similarly notes, “Map services and data downloaded from The National Map are free and in the public domain.” (*[*What are the Terms of Use/Licensing for map services and data from The National Map? | U.S. Geological Survey*](https://www.usgs.gov/faqs/what-are-terms-uselicensing-map-services-and-data-national-map)*). The NHDPlus and NCLD are distributed by the USGS and are considered public domain. The Pennsylvania Spatial Data Access notes on their website, “The purpose of PASDA is to serve as a comprehensive and coordinated open geospatial data portal that provides free public access to geospatial data and information by, for, and about the Commonwealth of Pennsylvania.” (*[*https://www.pasda.psu.edu/about.html#:~:text=Efficiency%2D%2DPASDA's%20streamlined%20processes,of%20charge%20to%20the%20public*](https://www.pasda.psu.edu/about.html#:~:text=Efficiency%2D%2DPASDA's%20streamlined%20processes,of%20charge%20to%20the%20public)*.) This clarification has been reflected in the figure caption.*

**Comment #5:** If the reviewer comments include a recommendation to cite specific previously published works, please review and evaluate these publications to determine whether they are relevant and should be cited. There is no requirement to cite these works unless the editor has indicated otherwise.

*Although the reviewer did not explicitly request that specific previously published works be cited, they noted several papers that may be relevant to our study. We carefully reviewed each of the suggested publications, evaluated their relevance to our objectives, and added citations where appropriate.*

**Response to Reviewer #1**

*We want to thank the reviewer for their insightful comments. They were very helpful and improved the manuscript. Please note the reviewer’s comments were not originally numbered; but for clarity and ease of reference, we have numbered them below and respond to each comment in turn.*

**Comment #1:** Firstly, the wide range of environmental covariates measured could have been analyzed further using principal components analysis and PERMANOVA. Standardized environmental data might show stronger combined effects between land management strategies than individually.

*We appreciate this suggestion and agree that a multivariate evaluation of environmental covariates is informative. To address this comment, we conducted the additional analyses recommended by the reviewer and added text to the to the manuscript reporting the results in the Methods and Results sections. We standardized all measured covariates and conducted a PCA and PERMANOVA with permutations constrained by watershed/ecoregion strata. Although the standardized environmental data showed stronger combined effects, the new analysis showed substantial overlap in multivariate environmental space and continued to show no significant difference between land sharing and land sparing streams (PERMANOVA: R² = 0.016, P = 0.17). These results confirm that no measured environmental conditions differed between site types, either individually or collectively, and therefore do not confound comparisons between land-use strategies. Thus, even when considered collectively, environmental conditions did not differ between land-use strategies. We have noted this in our methods and results sections (lines 257-263 & 303-310).*

**Comment #2:** The PERMANOVA used for testing macroinvertebrate composition could be improved by using the vegan package in R. Hellinger-transforming the data prior to applying the Bray-Curtis Dissimilarity would help reduce the influence of hyperabundant and rare taxa. Using “adonis2” in R could take advantage of permuting by Site pair to deal with the nested data and associated non-independencies.

*This was a helpful suggestion. In response, we reanalyzed the community composition data using the vegan package in R. Species abundances were Hellinger-transformed prior to calculating Bray–Curtis dissimilarities to reduce the influence of hyperabundant taxa while retaining information from rare taxa. We then used the adonis2 function with permutations restricted within matched stream pairs to account for the paired study design.*

*The revised analysis yielded qualitatively similar conclusions to our original analyses. Macroinvertebrate community composition did not differ significantly between sharing and sparing sites when permutations were restricted within site pairs (PERMANOVA: F₁,₈₀ = 1.26, R² = 0.015, p = 0.21). Multivariate dispersion did not differ between land-use types (p = 0.42), indicating that results were not driven by differences in within-group variability.*

*We have updated the Methods and Results sections with these new analyses and conclusions (see lines 264-275 & 331-338).*

**Comment #3:** I would like to know more about how functional feeding groups were treated prior to analysis. Using a Community Weighted Mean (CWM) trait values approach might be a more appropriate way to assess the functional composition of macroinvertebrate communities. This could help incorporate “fuzzy coding” since invertebrate are unlikely to fit neatly into a single FFG with feeding plasticity and ontogenetic diet shifts. The dbFD function in the FD R package can generate CWM trait values without too much trouble.

*We respectfully disagree that a Community Weighted Mean (CWM) framework with fuzzy coding is more appropriate for the objectives of this study. Our goal was to test whether land sharing and land sparing strategies differ in broad functional feeding group composition, as these coarse trophic categories are widely used indicators of ecosystem functioning in stream ecology. Taxa were therefore assigned to functional feeding groups based on their primary feeding mode following established and standardized classifications commonly applied in biomonitoring and comparative studies. Because our hypothesis concerned differences in overall trophic structure (e.g., relative abundance of shredders, scrapers, collectors, predators) rather than continuous variation in trait affinity or ontogenetic shifts, a categorical FFG framework directly addresses the research question. Incorporating fuzzy coding and CWM trait values would shift the analysis toward fine-scale trait weighting that is continuous, and which would likely obfuscate trends. For these reasons, we retained the categorical FFG approach.*

**Comment #4:** It could be quite easy for the authors to quantity the catchment context using land cover information. The result land cover could be decomposed into axes scores using PCA to generate control variables that help determine the potential for LSLS strategies to influence macroinvertebrate communities. Specifically I am thinking that upstream degradation might override the potential for more local-scale intervention to influence macroinvertebrate communities (sensu the fuzzy logic that Bernhardt and Palmer 2011 Ecol. Appl. outline).

*We appreciate the reviewer’s concern regarding potential catchment-scale influences; however, we have reason to believe this was not an issue in our study. Our paired study design was specifically structured to control for upstream land use. Streams within each pair were in close geographic proximity, within the same HUC-8 watershed and the same U.S. EPA Level III ecoregion. Because paired sites were separated by relatively short distances, they share nearly identical upstream land cover and catchment-scale conditions. Thus, any broad-scale watershed degradation or upstream land-use patterns would be expected to influence both streams within a pair similarly. For example, the clustered and dispersed sites within the Upper West Branch Susquehanna subbasin were approximately 17.9 miles apart, while the paired sites within the Lower Susquehanna–Penns subbasin were only 3.8 miles apart. These distances, combined with shared watershed and ecoregional boundaries, indicate that upstream context was effectively controlled by the paired design.*

**Comment #5:** L112 Where were the study sites located? It looks like Appalachian in Pennsylvania, but it is not explicitly stated here. How many ecoregions did the site pairs occupy?

*Thank you for pointing out that we failed to give a full description of the study location. In response, we have revised the manuscript to explicitly state the geographic location of the study sites and clarify the ecoregional context. Specifically, we now note that all study sites were located in central and western Pennsylvania, USA. Site pairs were distributed across three U.S. EPA Level III ecoregions (67, 69, and 70), representing the Ridge and Valley, Western Allegheny Plateau, and Central Appalachians (see lines 117-120).*

**Comment #6:** L117 Replace “…next:” with “below.”

*Corrected*

**Comment #7:** L188 Sediment is likely to be an important variable that influences macroinvertebrate communities. Could the authors consider deriving a % fine sediment metric from the field data (see Burdon et al. 2013 Ecol. Appl. for why deposited sediment could be of interest)?

*We thank the reviewer for this excellent suggestion and agree that fine sediment deposition is an important driver of macroinvertebrate community structure, as demonstrated by Burdon et al. (2013). In this study, we measured substrate size using a standard gravelometer following Wolman pebble-count procedures, which classifies particles into discrete size classes (e.g., 2, 32, 45, 64, 128, 180, and 256 mm). Although particles <2 mm are generally considered fine sediment, gravelometer-based pebble counts are not well suited for accurately quantifying the areal cover of deposited surface fines within riffles, as very small particles are difficult to capture and may be underrepresented. Consequently, we are unable to derive a reliable estimate of percent fine sediment cover from these data. We have clarified this methodological limitation in the Methods section (lines 201-205).*

**Comment #8:** L216 How many individuals were enumerated and identified using this method (i.e., mean, median, minimum, and maximum)?

*We have added summary statistics (i.e., mean, median, minimum, and maximum number of individuals per sample) to the Methods section (lines 237-238).*
