# Supplementary material for: Land sparing or sharing: A paired stream study to evaluate the relationship between land use configuration and macroinvertebrate diversity in streams
Source: PLoS One. 2026 Aug 12;21(8):e0354515. doi: 10.1371/journal.pone.0354515 (PMC13465839; doi:10.1371/journal.pone.0354515)
Supplement: Supplementary file 16 [file pone.0354515.s010.docx]

**S2–S2.1 Table. Linear Mixed Model (LMM) Estimates of Covariate Effects on Evenness Difference.** These tables show the effects of conductivity and phosphorus differences on evenness difference, with (S2) and without (S2.1) outliers, and indicate whether the observed differences in evenness were statistically significant.

Table2.

| **Variable** | **Estimate ± SE** | **T-Statistic** | **P-Value** |
| --- | --- | --- | --- |
| Evenness_dif (β_0_) | 0.67 ± 0.03 | 2.21 | **0.04^*^** |
| Cond_dif (β_1_) | -0.0002 ± 0.0001 | -1.49 | 0.14 |
| P_dif (β_2_) | -0.26 ± 0.14 | -0.81 | 0.42 |

Table2.1 outlier removed

| **Variable** | **Estimate ± SE** | **T-Statistic** | **P-Value** |
| --- | --- | --- | --- |
| Evenness_dif (β_0_) | 0.02 ± 0.015 | 1.37 | 0.19 |
| Cond_dif (β_1_) | 0.00001 ± 0.00003 | 0.22 | 0.83 |
| P_dif (β_2_) | 0.0001 ± 0.001 | 0.05 | 0.96 |
