# Supplementary material for: Land sparing or sharing: A paired stream study to evaluate the relationship between land use configuration and macroinvertebrate diversity in streams
Source: PLoS One. 2026 Aug 12;21(8):e0354515. doi: 10.1371/journal.pone.0354515 (PMC13465839; doi:10.1371/journal.pone.0354515)
Supplement: Supplementary file 18 [file pone.0354515.s012.docx]

**S4.1–S4.6 Table. Linear Mixed Model (LMM) Estimates of Covariate Effects on FFG Differences.** These tables show the effects of conductivity and phosphorus differences on functional feeding group (FFG) differences (Collector-filterers (S4.1), Collector-gatherers (S4.2), Herbivores (S4.3), Omnivores (S4.4), Predators (S4.5), Shredders (S4.6)), and indicate whether the observed differences in FFGs were statistically significant.

Table4.1 Collector-filterer

| **Variable** | **Estimate ± SE** | **T-Statistic** | **P-Value** |
| --- | --- | --- | --- |
| CF_dif (β_0_) | 6.11 **±** 2.40 | 2.55 | **0.02*** |
| Cond_dif (β_1_) | -0.02 **±** 0.01 | -1.90 | 0.06 |
| P_dif (β_2_) | 0.06 **±** 0.34 | 0.19 | 0.85 |

Table4.2 Collector-gatherer

| **Variable** | **Estimate ± SE** | **T-Statistic** | **P-Value** |
| --- | --- | --- | --- |
| CG_dif (β_0_) | -3.93 ± 2.58 | -1.52 | 0.15 |
| Cond_dif (β_1_) | 0.01 ± 0.01 | 1.13 | 0.26 |
| P_dif (β_2_) | -0.37 ± 0.27 | -1.34 | 0.19 |

Table4.3 Herbivore

| **Variable** | **Estimate ± SE** | **T-Statistic** | **P-Value** |
| --- | --- | --- | --- |
| Herb_dif (β_0_) | -2.68 ± 2.25 | -1.19 | 0.25 |
| Cond_dif (β_1_) | 0.003 ± 0.009 | 0.35 | 0.73 |
| P_dif (β_2_) | -0.18 ± 0.28 | -0.63 | 0.53 |

Table4.4 Omnivore

| **Variable** | **Estimate ± SE** | **T-Statistic** | **P-Value** |
| --- | --- | --- | --- |
| OM_dif (β_0_) | 0.08 ± 0.22 | 0.37 | 0.72 |
| Cond_dif (β_1_) | -0.0006 ± 0.0006 | -0.93 | 0.36 |
| P_dif (β_2_) | 0.003 ± 0.021 | 0.14 | 0.89 |

Table4.5 Predator

| **Variable** | **Estimate ± SE** | **T-Statistic** | **P-Value** |
| --- | --- | --- | --- |
| Pred_dif (β_0_) | 0.41 ± 0.31 | 1.29 | 0.21 |
| Cond_dif (β_1_) | -0.001 ± 0.001 | -1.14 | 0.26 |
| P_dif (β_2_) | -0.04 ± 0.03 | -1.17 | 0.25 |

Table4.6 Shredder

| **Variable** | **Estimate ± SE** | **T-Statistic** | **P-Value** |
| --- | --- | --- | --- |
| Shred_dif (β_0_) | 0.45 ± 0.33 | 1.37 | 0.19 |
| Cond_dif (β_1_) | -0.003 ± 0.001 | -2.27 | **0.03^*^** |
| P_dif (β_2_) | -0.02± 0.04 | -0.39 | 0.69 |
