# Supplementary material for: Land sparing or sharing: A paired stream study to evaluate the relationship between land use configuration and macroinvertebrate diversity in streams
Source: PLoS One. 2026 Aug 12;21(8):e0354515. doi: 10.1371/journal.pone.0354515 (PMC13465839; doi:10.1371/journal.pone.0354515)
Supplement: Supplementary file 20 [file pone.0354515.s015.docx]

May 30^th^, 2026

Re: Revision of manuscript PONE-D-25-45509

Dear Dr. Marina Vilencia,

Thank you again for the opportunity to revise our manuscript (PONE-D-25-45509). We greatly appreciate the time and effort invested by both the reviewer and the editorial team in evaluating our work.

We have carefully considered all comments and have incorporated nearly all of the suggested editorial revisions into the manuscript. We believe these changes have improved the clarity and overall quality of the paper.

There were a small number of comments that we were unable to fully accommodate, and our rationale for those decisions is provided in point by point responses below. Thank you again for your consideration of our manuscript. If you have any questions or concerns, we would be happy to address them. We look forward to your decision.

Sincerely,

Sugjit S. Padda, Ph.D.

Bradley J. Cardinale, Professor

**Comment #1:** Do you have climatic data for those regions? E.g. climate type (Koppen), mean annual percipitation and air temperature?

*We did not include separate climatic data for the ecoregions in this study. However, U.S. EPA Level III ecoregions are defined using combinations of climate, geology, topography, soils, vegetation, and land use characteristics (Woods et al., 1996). Consequently, the selected ecoregions inherently capture broad climatic variation across the study area. Because climate was not a focal variable in our analyses and the ecoregion framework already incorporates climatic differences, we did not include additional climate metrics.*

**Comment #2:** Where did you show the taxa list?

*The complete taxa list is provided in the “LSLS_Taxa_Data_Raw” file available in the Dryad repository associated with this manuscript.*

**Comment # 3:** Why didn't you calculate abundance as ind/m²?

*Sampling area was standardized across all stream reaches and sampling events, making abundance values directly comparable among sites. Converting abundance to individuals per square meter would therefore only change the scale of the response variable and would not alter the interpretation of the results. For simplicity and consistency with the original dataset, we retained abundance as raw counts.*

**Comment #4:** Where are the PCA results?

*We thank the editor for this comment. While the PCA code and associated data were already available in the Dryad repository, we agree that the PCA results should be more clearly presented within the manuscript materials. In response, we have added a summary table of PCA loadings and variance explained to the Supplementary Materials (Table S5), allowing readers to more readily evaluate and interpret the PCA results.*
