# Supplementary material for: Land sparing or sharing: A paired stream study to evaluate the relationship between land use configuration and macroinvertebrate diversity in streams
Source: PLoS One. 2026 Aug 12;21(8):e0354515. doi: 10.1371/journal.pone.0354515 (PMC13465839; doi:10.1371/journal.pone.0354515)
Supplement: Supplementary file 21 [file pone.0354515.s016.docx]

July 2^nd^, 2026

Re: Revision of manuscript PONE-D-25-45509

Dear Dr. Marina Vilencia,

Thank you again for the opportunity to revise our manuscript (PONE-D-25-45509). We greatly appreciate the time and effort invested by both the reviewer and the editorial team in evaluating our work.

This submission represents the fourth round of revisions. The comments provided during this round were primarily minor and editorial in nature. We carefully considered each comment and revised the manuscript accordingly, including clarifying the supplemental table legends and making numerous editorial improvements throughout the manuscript. We believe these revisions have further improved the clarity and presentation of our work without changing its scientific content or conclusions.

The only recommendation we did not adopt was the addition of a separate conclusion section. We respectfully chose not to make this change because it had not been requested during the previous rounds of review, and we believe the manuscript already concludes appropriately within the Discussion section.

We respectfully note that this will be the final round of revisions we are able to undertake for this manuscript. We hope that the revisions made throughout the review process have addressed the remaining concerns and that the manuscript is now suitable for publication. If additional substantive revisions are still considered necessary prior to acceptance, we would appreciate knowing that at this stage so that we may consider submitting the manuscript elsewhere.

Thank you again for your time and consideration. We appreciate your efforts throughout the review process and look forward to your decision.

Sincerely,

Sugjit S. Padda, Ph.D.

Bradley J. Cardinale, Professor
