# Supplementary material for: Land sparing or sharing: A paired stream study to evaluate the relationship between land use configuration and macroinvertebrate diversity in streams
Source: PLoS One. 2026 Aug 12;21(8):e0354515. doi: 10.1371/journal.pone.0354515 (PMC13465839; doi:10.1371/journal.pone.0354515)
Supplement: Peer review history

# Peer review: Decision Letter 0

**DOI:** 10.1371/journal.pone.0354515.r001

**Authors:** Marina Vilenica

9 Dec 2025

Dear Dr. Padda,

Please submit your revised manuscript by Jan 23 2026 11:59PM. If you will need more time than this to complete your revisions, please reply to this message or contact the journal office at plosone@plos.org. When you're ready to submit your revision, log on to https://www.editorialmanager.com/pone/ and select the 'Submissions Needing Revision' folder to locate your manuscript file.

- A rebuttal letter that responds to each point raised by the academic editor and reviewer(s). You should upload this letter as a separate file labeled 'Response to Reviewers'.
- A marked-up copy of your manuscript that highlights changes made to the original version. You should upload this as a separate file labeled 'Revised Manuscript with Track Changes'.
- An unmarked version of your revised paper without tracked changes. You should upload this as a separate file labeled 'Manuscript'.

If you would like to make changes to your financial disclosure, please include your updated statement in your cover letter. Guidelines for resubmitting your figure files are available below the reviewer comments at the end of this letter.

If applicable, we recommend that you deposit your laboratory protocols in protocols.io to enhance the reproducibility of your results. Protocols.io assigns your protocol its own identifier (DOI) so that it can be cited independently in the future. For instructions see: https://journals.plos.org/plosone/s/submission-guidelines#loc-laboratory-protocols. Additionally, PLOS ONE offers an option for publishing peer-reviewed Lab Protocol articles, which describe protocols hosted on protocols.io. Read more information on sharing protocols at https://plos.org/protocols?utm_medium=editorial-email&utm_source=authorletters&utm_campaign=protocols.

We look forward to receiving your revised manuscript.

Kind regards,

Marina Vilenica

Academic Editor

PLOS One

Journal Requirements:

When submitting your revision, we need you to address these additional requirements.

1. Please ensure that your manuscript meets PLOS ONE's style requirements, including those for file naming. The PLOS ONE style templates can be found at

https://journals.plos.org/plosone/s/file?id=wjVg/PLOSOne_formatting_sample_main_body.pdf and

https://journals.plos.org/plosone/s/file?id=ba62/PLOSOne_formatting_sample_title_authors_affiliations.pdf.

2. In your Methods section, please provide additional information regarding the permits you obtained for the work. Please ensure you have included the full name of the authority that approved the field site access and, if no permits were required, a brief statement explaining why.

3. Please note that your Data Availability Statement is currently missing [the repository name and/or the DOI/accession number of each dataset OR a direct link to access each database]. If your manuscript is accepted for publication, you will be asked to provide these details on a very short timeline. We therefore suggest that you provide this information now, though we will not hold up the peer review process if you are unable.

4. We note thatFigure 1 in your submission contain [map/satellite] images which may be copyrighted. All PLOS content is published under the Creative Commons Attribution License (CC BY 4.0), which means that the manuscript, images, and Supporting Information files will be freely available online, and any third party is permitted to access, download, copy, distribute, and use these materials in any way, even commercially, with proper attribution. For these reasons, we cannot publish previously copyrighted maps or satellite images created using proprietary data, such as Google software (Google Maps, Street View, and Earth). For more information, see our copyright guidelines: http://journals.plos.org/plosone/s/licenses-and-copyright.

We require you to either (1) present written permission from the copyright holder to publish these figures specifically under the CC BY 4.0 license, or (2) remove the figures from your submission:

a. You may seek permission from the original copyright holder of Figure 1 to publish the content specifically under the CC BY 4.0 license.

We recommend that you contact the original copyright holder with the Content Permission Form (http://journals.plos.org/plosone/s/file?id=7c09/content-permission-form.pdf) and the following text:

“I request permission for the open-access journal PLOS ONE to publish XXX under the Creative Commons Attribution License (CCAL) CC BY 4.0 (http://creativecommons.org/licenses/by/4.0/). Please be aware that this license allows unrestricted use and distribution, even commercially, by third parties. Please reply and provide explicit written permission to publish XXX under a CC BY license and complete the attached form.”

Please upload the completed Content Permission Form or other proof of granted permissions as an "Other" file with your submission.

In the figure caption of the copyrighted figure, please include the following text: “Reprinted from [ref] under a CC BY license, with permission from [name of publisher], original copyright [original copyright year].”

b. If you are unable to obtain permission from the original copyright holder to publish these figures under the CC BY 4.0 license or if the copyright holder’s requirements are incompatible with the CC BY 4.0 license, please either i) remove the figure or ii) supply a replacement figure that complies with the CC BY 4.0 license. Please check copyright information on all replacement figures and update the figure caption with source information. If applicable, please specify in the figure caption text when a figure is similar but not identical to the original image and is therefore for illustrative purposes only.

The following resources for replacing copyrighted map figures may be helpful:

USGS National Map Viewer (public domain): http://viewer.nationalmap.gov/viewer/

The Gateway to Astronaut Photography of Earth (public domain): http://eol.jsc.nasa.gov/sseop/clickmap/

Maps at the CIA (public domain): https://www.cia.gov/library/publications/the-world-factbook/index.html and https://www.cia.gov/library/publications/cia-maps-publications/index.html

NASA Earth Observatory (public domain): http://earthobservatory.nasa.gov/

Landsat: http://landsat.visibleearth.nasa.gov/

USGS EROS (Earth Resources Observatory and Science (EROS) Center) (public domain): http://eros.usgs.gov/#

Natural Earth (public domain): http://www.naturalearthdata.com/

5. If the reviewer comments include a recommendation to cite specific previously published works, please review and evaluate these publications to determine whether they are relevant and should be cited. There is no requirement to cite these works unless the editor has indicated otherwise.

Reviewers' comments:

Reviewer's Responses to Questions

**Comments to the Author**

1. Is the manuscript technically sound, and do the data support the conclusions?

Reviewer #1: Partly

**********

2. Has the statistical analysis been performed appropriately and rigorously?

Reviewer #1: No

**********

3. Have the authors made all data underlying the findings in their manuscript fully available?

The PLOS Data policy

Reviewer #1: Yes

**********

4. Is the manuscript presented in an intelligible fashion and written in standard English?

Reviewer #1: Yes

**********

Reviewer #1: This is a well-written study investigating the potential for LSLS strategies to influence stream macroinvertebrate communities. Whilst there have been extensive evaluations of LSLS strategies in terrestrial ecosystems, the same cannot be said for streams. This is despite the importance of surrounding land uses for stream biodiversity and functioning (“In every sense, the valley rules the stream”). Consequently, this study is novel in considering how LSLS strategies affect stream ecosystems.

The study is well-written and uses an appropriate design to test the effects of different land management strategies. However, I found that there could be improvements made to the data analysis which might reveal effects currently undetected:

Firstly, the wide range of environmental covariates measured could have been analyzed further using principal components analysis and PERMANOVA. Standardized environmental data might show stronger combined effects between land management strategies than individually.

The PERMANOVA used for testing macroinvertebrate composition could be improved by using the vegan package in R. Hellinger-transforming the data prior to applying the Bray-Curtis Dissimilarity would help reduce the influence of hyperabundant and rare taxa. Using “adonis2” in R could take advantage of permuting by Site pair to deal with the nested data and associated non-independencies.

I would like to know more about how functional feeding groups were treated prior to analysis. Using a Community Weighted Mean (CWM) trait values approach might be a more appropriate way to assess the functional composition of macroinvertebrate communities. This could help incorporate “fuzzy coding” since invertebrate are unlikely to fit neatly into a single FFG with feeding plasticity and ontogenetic diet shifts. The dbFD function in the FD R package can generate CWM trait values without too much trouble.

It could be quite easy for the authors to quantity the catchment context using land cover information. The result land cover could be decomposed into axes scores using PCA to generate control variables that help determine the potential for LSLS strategies to influence macroinvertebrate communities. Specifically I am thinking that upstream degradation might override the potential for more local-scale intervention to influence macroinvertebrate communities (sensu the fuzzy logic that Bernhardt and Palmer 2011 Ecol. Appl. outline).

Minor comments:

L112 Where were the study sites located? It looks like Appalachian in Pennsylvania, but it is not explicitly stated here. How many ecoregions did the site pairs occupy?

L117 Replace “…next:” with “below.”

L188 Sediment is likely to be an important variable that influences macroinvertebrate communities. Could the authors consider deriving a % fine sediment metric from the field data (see Burdon et al. 2013 Ecol. Appl. for why deposited sediment could be of interest)?

L216 How many individuals were enumerated and identified using this method (i.e., mean, median, minimum, and maximum)?

**********

what does this mean?). If published, this will include your full peer review and any attached files.

If you choose “no”, your identity will remain anonymous but your review may still be made public.

**Do you want your identity to be public for this peer review?**  For information about this choice, including consent withdrawal, please see our Privacy Policy

Reviewer #1: No

**********

[NOTE: If reviewer comments were submitted as an attachment file, they will be attached to this email and accessible via the submission site. Please log into your account, locate the manuscript record, and check for the action link "View Attachments". If this link does not appear, there are no attachment files.]

To ensure your figures meet our technical requirements, please review our figure guidelines: https://journals.plos.org/plosone/s/figures

You may also use PLOS’s free figure tool, NAAS, to help you prepare publication quality figures: https://journals.plos.org/plosone/s/figures#loc-tools-for-figure-preparation.

NAAS will assess whether your figures meet our technical requirements by comparing each figure against our figure specifications.

# Author comment: Author response to Decision Letter 1

**DOI:** 10.1371/journal.pone.0354515.r002

23 Feb 2026

Response to Academic Editor:

Comment #1: Please ensure that your manuscript meets PLOS ONE's style requirements, including those for file naming.

We have carefully revised the manuscript to ensure full compliance with PLOS ONE style requirements, including formatting and file naming conventions.

Comment #2: In your Methods section, please provide additional information regarding the permits you obtained for the work. Please ensure you have included the full name of the authority that approved the field site access and, if no permits were required, a brief statement explaining why.

We did not require state or federal permits for macroinvertebrate sampling because all collections were conducted at publicly accessible bridge crossings from public rights-of-way. Sampling did not involve restricted waterways or access to private property. We have clarified this in the Methods section to explicitly state that no permits were required and to identify the nature of site access (lines 186-188).

Comment #3: Please note that your Data Availability Statement is currently missing [the repository name and/or the DOI/accession number of each dataset OR a direct link to access each database]. If your manuscript is accepted for publication, you will be asked to provide these details on a very short timeline. We therefore suggest that you provide this information now, though we will not hold up the peer review process if you are unable.

All data and associated R code has been submitted to Dryad. The DOI identifier associated with the manuscript data is (https://doi.org/10.5061/dryad.b8gtht7t0, and can be accessed temporarily through this link: http://datadryad.org/share/LINK_NOT_FOR_PUBLICATION/MtSAUGD-GII7qDemzlz0rD3_wi_04Q0yteFEXgSKzOY). The data will remain private for the peer review process and will be made public available after submission. The data availability statement can be found after the abstract in the manuscript (lines 47-50).

Comment #4: We note that Figure 1 in your submission contain [map/satellite] images which may be copyrighted. All PLOS content is published under the Creative Commons Attribution License (CC BY 4.0), which means that the manuscript, images, and Supporting Information files will be freely available online, and any third party is permitted to access, download, copy, distribute, and use these materials in any way, even commercially, with proper attribution. For these reasons, we cannot publish previously copyrighted maps or satellite images created using proprietary data, such as Google software (Google Maps, Street View, and Earth).

Figure 1 was created entirely using publicly available U.S. federal government geospatial datasets (EPA Level III Ecoregions, USGS HUC-8 subbasins, NHDPlus stream networks, NLCD land cover, and Pennsylvania Spatial Data Access). No proprietary basemaps (e.g., Google Maps, ESRI World Imagery, or other copyrighted satellite imagery) were used. All datasets are in the public domain and compatible with publication under a CC BY 4.0 license. The EPA states, “Unless otherwise specified, geospatial data produced by the EPA is by default in the public domain and is not subject to domestic copyright protection under 17 U.S.C. § 105.” (EPA Disclaimers | US EPA). The USGS similarly notes, “Map services and data downloaded from The National Map are free and in the public domain.” (What are the Terms of Use/Licensing for map services and data from The National Map? | U.S. Geological Survey). The NHDPlus and NCLD are distributed by the USGS and are considered public domain. The Pennsylvania Spatial Data Access notes on their website, “The purpose of PASDA is to serve as a comprehensive and coordinated open geospatial data portal that provides free public access to geospatial data and information by, for, and about the Commonwealth of Pennsylvania.” (https://www.pasda.psu.edu/about.html#:~:text=Efficiency%2D%2DPASDA's%20streamlined%20processes,of%20charge%20to%20the%20public.) This clarification has been reflected in the figure caption.

Comment #5: If the reviewer comments include a recommendation to cite specific previously published works, please review and evaluate these publications to determine whether they are relevant and should be cited. There is no requirement to cite these works unless the editor has indicated otherwise.

Although the reviewer did not explicitly request that specific previously published works be cited, they noted several papers that may be relevant to our study. We carefully reviewed each of the suggested publications, evaluated their relevance to our objectives, and added citations where appropriate.

Response to Reviewer #1

We want to thank the reviewer for their insightful comments. They were very helpful and improved the manuscript. Please note the reviewer’s comments were not originally numbered; but for clarity and ease of reference, we have numbered them below and respond to each comment in turn.

Comment #1: Firstly, the wide range of environmental covariates measured could have been analyzed further using principal components analysis and PERMANOVA. Standardized environmental data might show stronger combined effects between land management strategies than individually.

We appreciate this suggestion and agree that a multivariate evaluation of environmental covariates is informative. To address this comment, we conducted the additional analyses recommended by the reviewer and added text to the to the manuscript reporting the results in the Methods and Results sections. We standardized all measured covariates and conducted a PCA and PERMANOVA with permutations constrained by watershed/ecoregion strata. Although the standardized environmental data showed stronger combined effects, the new analysis showed substantial overlap in multivariate environmental space and continued to show no significant difference between land sharing and land sparing streams (PERMANOVA: R² = 0.016, P = 0.17). These results confirm that no measured environmental conditions differed between site types, either individually or collectively, and therefore do not confound comparisons between land-use strategies. Thus, even when considered collectively, environmental conditions did not differ between land-use strategies. We have noted this in our methods and results sections (lines 257-263 & 303-310).

Comment #2: The PERMANOVA used for testing macroinvertebrate composition could be improved by using the vegan package in R. Hellinger-transforming the data prior to applying the Bray-Curtis Dissimilarity would help reduce the influence of hyperabundant and rare taxa. Using “adonis2” in R could take advantage of permuting by Site pair to deal with the nested data and associated non-independencies.

This was a helpful suggestion. In response, we reanalyzed the community composition data using the vegan package in R. Species abundances were Hellinger-transformed prior to calculating Bray–Curtis dissimilarities to reduce the influence of hyperabundant taxa while retaining information from rare taxa. We then used the adonis2 function with permutations restricted within matched stream pairs to account for the paired study design.

The revised analysis yielded qualitatively similar conclusions to our original analyses. Macroinvertebrate community composition did not differ significantly between sharing and sparing sites when permutations were restricted within site pairs (PERMANOVA: F₁,₈₀ = 1.26, R² = 0.015, p = 0.21). Multivariate dispersion did not differ between land-use types (p = 0.42), indicating that results were not driven by differences in within-group variability.

We have updated the Methods and Results sections with these new analyses and conclusions (see lines 264-275 & 331-338).

Comment #3: I would like to know more about how functional feeding groups were treated prior to analysis. Using a Community Weighted Mean (CWM) trait values approach might be a more appropriate way to assess the functional composition of macroinvertebrate communities. This could help incorporate “fuzzy coding” since invertebrate are unlikely to fit neatly into a single FFG with feeding plasticity and ontogenetic diet shifts. The dbFD function in the FD R package can generate CWM trait values without too much trouble.

We respectfully disagree that a Community Weighted Mean (CWM) framework with fuzzy coding is more appropriate for the objectives of this study. Our goal was to test whether land sharing and land sparing strategies differ in broad functional feeding group composition, as these coarse trophic categories are widely used indicators of ecosystem functioning in stream ecology. Taxa were therefore assigned to functional feeding groups based on their primary feeding mode following established and standardized classifications commonly applied in biomonitoring and comparative studies. Because our hypothesis concerned differences in overall trophic structure (e.g., relative abundance of shredders, scrapers, collectors, predators) rather than continuous variation in trait affinity or ontogenetic shifts, a categorical FFG framework directly addresses the research question. Incorporating fuzzy coding and CWM trait values would shift the analysis toward fine-scale trait weighting that is continuous, and which would likely obfuscate trends. For these reasons, we retained the categorical FFG approach.

Comment #4: It could be quite easy for the authors to quantity the catchment context using land cover information. The result land cover could be decomposed into axes scores using PCA to generate control variables that help determine the potential for LSLS strategies to influence macroinvertebrate communities. Specifically I am thinking that upstream degradation might override the potential for more local-scale intervention to influence macroinvertebrate communities (sensu the fuzzy logic that Bernhardt and Palmer 2011 Ecol. Appl. outline).

We appreciate the reviewer’s concern regarding potential catchment-scale influences; however, we have reason to believe this was not an issue in our study. Our paired study design was specifically structured to control for upstream land use. Streams within each pair were in close geographic proximity, within the same HUC-8 watershed and the same U.S. EPA Level III ecoregion. Because paired sites were separated by relatively short distances, they share nearly identical upstream land cover and catchment-scale conditions. Thus, any broad-scale watershed degradation or upstream land-use patterns would be expected to influence both streams within a pair similarly. For example, the clustered and dispersed sites within the Upper West Branch Susquehanna subbasin were approximately 17.9 miles apart, while the paired sites within the Lower Susquehanna–Penns subbasin were only 3.8 miles apart. These distances, combined with shared watershed and ecoregional boundaries, indicate that upstream context was effectively controlled by the paired design.

Comment #5: L112 Where were the study sites located? It looks like Appalachian in Pennsylvania, but it is not explicitly stated here. How many ecoregions did the site pairs occupy?

Thank you for pointing out that we failed to give a full description of the study location. In response, we have revised the manuscript to explicitly state the geographic location of the study sites and clarify the ecoregional context. Specifically, we now note that all study sites were located in central and western Pennsylvania, USA. Site pairs were distributed across three U.S. EPA Level III ecoregions (67, 69, and 70), representing the Ridge and Valley, Western Allegheny Plateau, and Central Appalachians (see lines 117-120).

Comment #6: L117 Replace “…next:” with “below.”

Corrected

Comment #7: L188 Sediment is likely to be an important variable that influences macroinvertebrate communities. Could the authors consider deriving a % fine sediment metric from the field data (see Burdon et al. 2013 Ecol. Appl. for why deposited sediment could be of interest)?

We thank the reviewer for this excellent suggestion and agree that fine sediment deposition is an important driver of macroinvertebrate community structure, as demonstrated by Burdon et al. (2013). In this study, we measured substrate size using a standard gravelometer following Wolman pebble-count procedures, which classifies particles into discrete size classes (e.g., 2, 32, 45, 64, 128, 180, and 256 mm). Although particles <2 mm are generally considered fine sediment, gravelometer-based pebble counts are not well suited for accurately quantifying the areal cover of deposited surface fines within riffles, as very small particles are difficult to capture and may be underrepresented. Consequently, we are unable to derive a reliable estimate of percent fine sediment cover from these data. We have clarified this methodological limitation in the Methods section (lines 201-205).

Comment #8: L216 How many individuals were enumerated and identified using this method (i.e., mean, median, minimum, and maximum)?

We have added summary statistics (i.e., mean, median, minimum, and maximum number of individuals per sample) to the Methods section (lines 237-238).

Attachment Submitted filename: Rebuttal_letter_Padda_Cardinale .docx

# Peer review: Decision Letter 1

**DOI:** 10.1371/journal.pone.0354515.r003

**Authors:** Marina Vilenica

11 Mar 2026

Dear Dr. Padda,

Thank you for submitting your manuscript to PLOS ONE. After careful consideration, we feel that it has merit but does not fully meet PLOS ONE’s publication criteria as it currently stands. Therefore, we invite you to submit a revised version of the manuscript that addresses the points raised during the review process.

Please submit your revised manuscript by Apr 25 2026 11:59PM. If you will need more time than this to complete your revisions, please reply to this message or contact the journal office at plosone@plos.org. When you're ready to submit your revision, log on to https://www.editorialmanager.com/pone/ and select the 'Submissions Needing Revision' folder to locate your manuscript file.

- A letter that responds to each point raised by the academic editor and reviewer(s). You should upload this letter as a separate file labeled 'Response to Reviewers'.
- A marked-up copy of your manuscript that highlights changes made to the original version. You should upload this as a separate file labeled 'Revised Manuscript with Track Changes'.
- An unmarked version of your revised paper without tracked changes. You should upload this as a separate file labeled 'Manuscript'.

If applicable, we recommend that you deposit your laboratory protocols in protocols.io to enhance the reproducibility of your results. Protocols.io assigns your protocol its own identifier (DOI) so that it can be cited independently in the future. For instructions see: https://journals.plos.org/plosone/s/submission-guidelines#loc-laboratory-protocols. Additionally, PLOS ONE offers an option for publishing peer-reviewed Lab Protocol articles, which describe protocols hosted on protocols.io. Read more information on sharing protocols at https://plos.org/protocols?utm_medium=editorial-email&utm_source=authorletters&utm_campaign=protocols.

We look forward to receiving your revised manuscript.

Kind regards,

Marina Vilenica

Academic Editor

PLOS One

Journal Requirements:

If the reviewer comments include a recommendation to cite specific previously published works, please review and evaluate these publications to determine whether they are relevant and should be cited. There is no requirement to cite these works unless the editor has indicated otherwise.

Please review your reference list to ensure that it is complete and correct. If you have cited papers that have been retracted, please include the rationale for doing so in the manuscript text, or remove these references and replace them with relevant current references. Any changes to the reference list should be mentioned in the rebuttal letter that accompanies your revised manuscript. If you need to cite a retracted article, indicate the article’s retracted status in the References list and also include a citation and full reference for the retraction notice.

Additional Editor Comments:

Dear authors, many thanks for improvements you made in your manuscript, in accordance with Reviewer's suggestions. The reviewer provided only minor suggestions, which I invite you to consider before your work can be accepted for publication.

[Note: HTML markup is below. Please do not edit.]

Reviewers' comments:

Reviewer's Responses to Questions

**Comments to the Author**

Reviewer #1: (No Response)

**********

2. Is the manuscript technically sound, and do the data support the conclusions?

Reviewer #1: Yes

**********

3. Has the statistical analysis been performed appropriately and rigorously?

Reviewer #1: Yes

**********

4. Have the authors made all data underlying the findings in their manuscript fully available?

The PLOS Data policy

Reviewer #1: Yes

**********

5. Is the manuscript presented in an intelligible fashion and written in standard English?

Reviewer #1: Yes

**********

Reviewer #1: I appreciate the consideration shown by the authors for my suggestions on ways to improve their manuscript. There are clear improvements in the rigor of the work where changes have been made. I don't have any further major comments other than some suggested minor corrections.

At L278 conductivity and phosphate concentrations are mentioned as covariates, presumably for the same logic described at L255. Making it explicitly clear at either instance of why conductivity and phosphate concentrations are used as covariates would help avoid any confusion for the reader. I expect that the procedure explained at L240-248 helps explain why Moran’s I was not included as a covariate in the GLMs despite differing between land use strategies.

The results of the PERMANOVA model testing macroinvertebrate species composition are duplicated at L331 and L337. The sentence at L335 is redundant here and could be moved/merged with the data analysis methods.

**********

what does this mean?). If published, this will include your full peer review and any attached files.

If you choose “no”, your identity will remain anonymous but your review may still be made public.

**Do you want your identity to be public for this peer review?**  For information about this choice, including consent withdrawal, please see our Privacy Policy

Reviewer #1: No

**********

[NOTE: If reviewer comments were submitted as an attachment file, they will be attached to this email and accessible via the submission site. Please log into your account, locate the manuscript record, and check for the action link "View Attachments". If this link does not appear, there are no attachment files.]

To ensure your figures meet our technical requirements, please review our figure guidelines: https://journals.plos.org/plosone/s/figures

You may also use PLOS’s free figure tool, NAAS, to help you prepare publication quality figures: https://journals.plos.org/plosone/s/figures#loc-tools-for-figure-preparation.

NAAS will assess whether your figures meet our technical requirements by comparing each figure against our figure specifications.

# Author comment: Author response to Decision Letter 2

**DOI:** 10.1371/journal.pone.0354515.r004

20 Apr 2026

Dear Dr. Marina Vilencia,

Thank you again for the opportunity to revise our manuscript PONE-D-25-45509 and for the constructive feedback provided by the reviewer. We have addressed the minor comments raised by the reviewer. Additionally, we have reviewed the reference list to assure that none of the works have been retracted.

Following this letter, we provide a point-by-point response explaining how we have addressed each reviewer comment. If you have any questions, concerns, or further requests for revision, we would be happy to address them. We look forward to your decision on this manuscript.

Sincerely,

Sugjit S. Padda, Ph.D.

Bradley J. Cardinale, Professor

Response to Reviewer #1

We want to thank the reviewer for their insightful comments. They were very helpful and improved the manuscript. Please note the reviewer’s comments were not originally numbered; but for clarity and ease of reference, we have numbered them below and respond to each comment in turn.

Comment #1: At L278 conductivity and phosphate concentrations are mentioned as covariates, presumably for the same logic described at L255. Making it explicitly clear at either instance of why conductivity and phosphate concentrations are used as covariates would help avoid any confusion for the reader. I expect that the procedure explained at L240-248 helps explain why Moran’s I was not included as a covariate in the GLMs despite differing between land use strategies.

We have revised the Methods to explicitly clarify the rationale for including conductivity and phosphate as covariates, ensuring consistency with the earlier explanation and improving clarity for the reader (lines 279-281).

Comment #2: The results of the PERMANOVA model testing macroinvertebrate species composition are duplicated at L331 and L337. The sentence at L335 is redundant here and could be moved/merged with the data analysis methods.

We have removed the duplicate PERMANOVA results and revised the text to eliminate the redundancy (lines 333-337).

Attachment Submitted filename: Response to Reviewers.docx

# Editor report: Decision Letter 2

**DOI:** 10.1371/journal.pone.0354515.r005

**Authors:** Marina Vilenica

27 Apr 2026

Thank you for submitting your manuscript to PLOS ONE. After careful consideration, we feel that it has merit but does not fully meet PLOS ONE’s publication criteria as it currently stands. Therefore, we invite you to submit a revised version of the manuscript that addresses the points raised during the review process.

==============================

**ACADEMIC EDITOR:**

==============================

Please submit your revised manuscript by Jun 11 2026 11:59PM. If you will need more time than this to complete your revisions, please reply to this message or contact the journal office at plosone@plos.org. When you're ready to submit your revision, log on to https://www.editorialmanager.com/pone/ and select the 'Submissions Needing Revision' folder to locate your manuscript file.

- A letter that responds to each point raised by the academic editor and reviewer(s). You should upload this letter as a separate file labeled 'Response to Reviewers'.
- A marked-up copy of your manuscript that highlights changes made to the original version. You should upload this as a separate file labeled 'Revised Manuscript with Track Changes'.
- An unmarked version of your revised paper without tracked changes. You should upload this as a separate file labeled 'Manuscript'.

If you would like to make changes to your financial disclosure, please include your updated statement in your cover letter. Guidelines for resubmitting your figure files are available below the reviewer comments at the end of this letter.

If applicable, we recommend that you deposit your laboratory protocols in protocols.io to enhance the reproducibility of your results. Protocols.io assigns your protocol its own identifier (DOI) so that it can be cited independently in the future. For instructions see: https://journals.plos.org/plosone/s/submission-guidelines#loc-laboratory-protocols. Additionally, PLOS ONE offers an option for publishing peer-reviewed Lab Protocol articles, which describe protocols hosted on protocols.io. Read more information on sharing protocols at https://plos.org/protocols?utm_medium=editorial-email&utm_source=authorletters&utm_campaign=protocols.

As the corresponding author, your ORCID iD is verified in the submission system and will appear in the published article. PLOS supports the use of ORCID, and we encourage all coauthors to register for an ORCID iD and use it as well. Please encourage your coauthors to verify their ORCID iD within the submission system before final acceptance, as unverified ORCID iDs will not appear in the published article. *Only*  the individual author can complete the verification step; PLOS staff *cannot*  verify ORCID iDs on behalf of authors.

We look forward to receiving your revised manuscript.

Kind regards,

Marina Vilenica

Academic Editor

PLOS One

Journal Requirements:

1. If the reviewer comments include a recommendation to cite specific previously published works, please review and evaluate these publications to determine whether they are relevant and should be cited. There is no requirement to cite these works unless the editor has indicated otherwise.

2. Please review your reference list to ensure that it is complete and correct. If you have cited papers that have been retracted, please include the rationale for doing so in the manuscript text, or remove these references and replace them with relevant current references. Any changes to the reference list should be mentioned in the rebuttal letter that accompanies your revised manuscript. If you need to cite a retracted article, indicate the article’s retracted status in the References list and also include a citation and full reference for the retraction notice.

[Note: HTML markup is below. Please do not edit.]

[NOTE: If reviewer comments were submitted as an attachment file, they will be attached to this email and accessible via the submission site. Please log into your account, locate the manuscript record, and check for the action link "View Attachments". If this link does not appear, there are no attachment files.]

To ensure your figures meet our technical requirements, please review our figure guidelines: https://journals.plos.org/plosone/s/figures

You may also use PLOS’s free figure tool, NAAS, to help you prepare publication quality figures: https://journals.plos.org/plosone/s/figures#loc-tools-for-figure-preparation.

NAAS will assess whether your figures meet our technical requirements by comparing each figure against our figure specifications.

Attachment Submitted filename: Manuscript_ED.docx

Attachment Submitted filename: S1_Table_ED.docx

Attachment Submitted filename: S2_Table_ED.docx

Attachment Submitted filename: S3_Table_ED.docx

Attachment Submitted filename: S4_Table_ED.docx

# Author comment: Author response to Decision Letter 3

**DOI:** 10.1371/journal.pone.0354515.r006

3 Jun 2026

Dear Dr. Marina Vilencia,

Thank you again for the opportunity to revise our manuscript (PONE-D-25-45509). We greatly appreciate the time and effort invested by both the reviewer and the editorial team in evaluating our work.

We have carefully considered all comments and have incorporated nearly all of the suggested editorial revisions into the manuscript. We believe these changes have improved the clarity and overall quality of the paper.

There were a small number of comments that we were unable to fully accommodate, and our rationale for those decisions is provided in point by point responses below. Thank you again for your consideration of our manuscript. If you have any questions or concerns, we would be happy to address them. We look forward to your decision.

Sincerely,

Sugjit S. Padda, Ph.D.

Bradley J. Cardinale, Professor

Comment #1: Do you have climatic data for those regions? E.g. climate type (Koppen), mean annual percipitation and air temperature?

We did not include separate climatic data for the ecoregions in this study. However, U.S. EPA Level III ecoregions are defined using combinations of climate, geology, topography, soils, vegetation, and land use characteristics (Woods et al., 1996). Consequently, the selected ecoregions inherently capture broad climatic variation across the study area. Because climate was not a focal variable in our analyses and the ecoregion framework already incorporates climatic differences, we did not include additional climate metrics.

Comment #2: Where did you show the taxa list?

The complete taxa list is provided in the “LSLS_Taxa_Data_Raw” file available in the Dryad repository associated with this manuscript.

Comment # 3: Why didn't you calculate abundance as ind/m²?

Sampling area was standardized across all stream reaches and sampling events, making abundance values directly comparable among sites. Converting abundance to individuals per square meter would therefore only change the scale of the response variable and would not alter the interpretation of the results. For simplicity and consistency with the original dataset, we retained abundance as raw counts.

Comment #4: Where are the PCA results?

We thank the editor for this comment. While the PCA code and associated data were already available in the Dryad repository, we agree that the PCA results should be more clearly presented within the manuscript materials. In response, we have added a summary table of PCA loadings and variance explained to the Supplementary Materials (Table S5), allowing readers to more readily evaluate and interpret the PCA results.

Attachment Submitted filename: Response_to_Reviewers_auresp_3.docx

# Editor report: Decision Letter 3

**DOI:** 10.1371/journal.pone.0354515.r007

**Authors:** Marina Vilenica

8 Jun 2026

Dear Dr. Padda,

Thank you for submitting your manuscript to PLOS ONE. After careful consideration, we feel that it has merit but does not fully meet PLOS ONE’s publication criteria as it currently stands. Therefore, we invite you to submit a revised version of the manuscript that addresses the points raised during the review process.

Please submit your revised manuscript by Jul 23 2026 11:59PM. If you will need more time than this to complete your revisions, please reply to this message or contact the journal office at plosone@plos.org. When you're ready to submit your revision, log on to https://www.editorialmanager.com/pone/ and select the 'Submissions Needing Revision' folder to locate your manuscript file.

- A letter that responds to each point raised by the academic editor and reviewer(s). You should upload this letter as a separate file labeled 'Response to Reviewers'.
- A marked-up copy of your manuscript that highlights changes made to the original version. You should upload this as a separate file labeled 'Revised Manuscript with Track Changes'.
- An unmarked version of your revised paper without tracked changes. You should upload this as a separate file labeled 'Manuscript'.

If you would like to make changes to your financial disclosure, please include your updated statement in your cover letter. Guidelines for resubmitting your figure files are available below the reviewer comments at the end of this letter.

If applicable, we recommend that you deposit your laboratory protocols in protocols.io to enhance the reproducibility of your results. Protocols.io assigns your protocol its own identifier (DOI) so that it can be cited independently in the future. For instructions see: https://journals.plos.org/plosone/s/submission-guidelines#loc-laboratory-protocols. Additionally, PLOS ONE offers an option for publishing peer-reviewed Lab Protocol articles, which describe protocols hosted on protocols.io. Read more information on sharing protocols at https://plos.org/protocols?utm_medium=editorial-email&utm_source=authorletters&utm_campaign=protocols.

As the corresponding author, your ORCID iD is verified in the submission system and will appear in the published article. PLOS supports the use of ORCID, and we encourage all coauthors to register for an ORCID iD and use it as well. Please encourage your coauthors to verify their ORCID iD within the submission system before final acceptance, as unverified ORCID iDs will not appear in the published article. *Only*  the individual author can complete the verification step; PLOS staff *cannot*  verify ORCID iDs on behalf of authors.

We look forward to receiving your revised manuscript.

Kind regards,

Marina Vilenica

Academic Editor

PLOS One

Journal Requirements:

If the reviewer comments include a recommendation to cite specific previously published works, please review and evaluate these publications to determine whether they are relevant and should be cited. There is no requirement to cite these works unless the editor has indicated otherwise.

Please review your reference list to ensure that it is complete and correct. If you have cited papers that have been retracted, please include the rationale for doing so in the manuscript text, or remove these references and replace them with relevant current references. Any changes to the reference list should be mentioned in the rebuttal letter that accompanies your revised manuscript. If you need to cite a retracted article, indicate the article’s retracted status in the References list and also include a citation and full reference for the retraction notice.

Additional Editor Comments:

[Note: HTML markup is below. Please do not edit.]

[NOTE: If reviewer comments were submitted as an attachment file, they will be attached to this email and accessible via the submission site. Please log into your account, locate the manuscript record, and check for the action link "View Attachments". If this link does not appear, there are no attachment files.]

To ensure your figures meet our technical requirements, please review our figure guidelines: https://journals.plos.org/plosone/s/figures

You may also use PLOS’s free figure tool, NAAS, to help you prepare publication quality figures: https://journals.plos.org/plosone/s/figures#loc-tools-for-figure-preparation.

NAAS will assess whether your figures meet our technical requirements by comparing each figure against our figure specifications.

Attachment Submitted filename: Manuscript (1)_MV.docx

# Author comment: Author response to Decision Letter 4

**DOI:** 10.1371/journal.pone.0354515.r008

8 Jul 2026

Dear Dr. Marina Vilencia,

Thank you again for the opportunity to revise our manuscript (PONE-D-25-45509). We greatly appreciate the time and effort invested by both the reviewer and the editorial team in evaluating our work.

This submission represents the fourth round of revisions. The comments provided during this round were primarily minor and editorial in nature. We carefully considered each comment and revised the manuscript accordingly, including clarifying the supplemental table legends and making numerous editorial improvements throughout the manuscript. We believe these revisions have further improved the clarity and presentation of our work without changing its scientific content or conclusions.

The only recommendation we did not adopt was the addition of a separate conclusion section. We respectfully chose not to make this change because it had not been requested during the previous rounds of review, and we believe the manuscript already concludes appropriately within the Discussion section.

We respectfully note that this will be the final round of revisions we are able to undertake for this manuscript. We hope that the revisions made throughout the review process have addressed the remaining concerns and that the manuscript is now suitable for publication. If additional substantive revisions are still considered necessary prior to acceptance, we would appreciate knowing that at this stage so that we may consider submitting the manuscript elsewhere.

Thank you again for your time and consideration. We appreciate your efforts throughout the review process and look forward to your decision.

Sincerely,

Sugjit S. Padda, Ph.D.

Bradley J. Cardinale, Professor

Attachment Submitted filename: Response_to_Reviewers_auresp_4.docx

# Editor report: Decision Letter 4

**DOI:** 10.1371/journal.pone.0354515.r009

**Authors:** Marina Vilenica

10 Jul 2026

Land sparing or sharing: A paired stream study to evaluate the relationship between land use configuration and macroinvertebrate diversity in streams.

PONE-D-25-45509R4

Dear Dr. Padda,

We’re pleased to inform you that your manuscript has been judged scientifically suitable for publication and will be formally accepted for publication once it meets all outstanding technical requirements.

Within one week, you’ll receive an e-mail detailing the required amendments. When these have been addressed, you’ll receive a formal acceptance letter and your manuscript will be scheduled for publication.

An invoice will be generated when your article is formally accepted. Please note, if your institution has a publishing partnership with PLOS and your article meets the relevant criteria, all or part of your publication costs will be covered. Please make sure your user information is up-to-date by logging into Editorial Manager at Editorial Manager® and clicking the ‘Update My Information' link at the top of the page. For questions related to billing, please contact billing support.

If your institution or institutions have a press office, please notify them about your upcoming paper to help maximize its impact. If they’ll be preparing press materials, please inform our press team as soon as possible -- no later than 48 hours after receiving the formal acceptance. Your manuscript will remain under strict press embargo until 2 pm Eastern Time on the date of publication. For more information, please contact onepress@plos.org.

Kind regards,

Marina Vilenica

Academic Editor

PLOS One

Additional Editor Comments (optional):

Reviewers' comments:

# Editor report: Acceptance letter

**DOI:** 10.1371/journal.pone.0354515.r010

**Authors:** Marina Vilenica

PONE-D-25-45509R4

PLOS One

Dear Dr. Padda,

I'm pleased to inform you that your manuscript has been deemed suitable for publication in PLOS One. Congratulations! Your manuscript is now being handed over to our production team.

At this stage, our production department will prepare your paper for publication. This includes ensuring the following:

* All references, tables, and figures are properly cited

* All relevant supporting information is included in the manuscript submission,

* There are no issues that prevent the paper from being properly typeset

You will receive further instructions from the production team, including instructions on how to review your proof when it is ready. Please keep in mind that we are working through a large volume of accepted articles, so please give us a few days to review your paper and let you know the next and final steps.

Lastly, if your institution or institutions have a press office, please let them know about your upcoming paper now to help maximize its impact. If they'll be preparing press materials, please inform our press team within the next 48 hours. Your manuscript will remain under strict press embargo until 2 pm Eastern Time on the date of publication. For more information, please contact onepress@plos.org.

You will receive an invoice from PLOS for your publication fee after your manuscript has reached the completed accept phase. If you receive an email requesting payment before acceptance or for any other service, this may be a phishing scheme. Learn how to identify phishing emails and protect your accounts at https://explore.plos.org/phishing.

If we can help with anything else, please email us at customercare@plos.org.

Thank you for submitting your work to PLOS ONE and supporting open access.

Kind regards,

PLOS ONE Editorial Office Staff

on behalf of

Dr. Marina Vilenica

Academic Editor

PLOS One
